# Interprofessional sense-making in the emergency department: A SenseMaker study

**DOI:** 10.1371/journal.pone.0282307

**Published:** 2023-03-09

**Authors:** Charmaine Cunningham, Marietjie Vosloo, Lee A. Wallis

**Affiliations:** 1 Division of Emergency Medicine, The University of Cape Town, Cape Town, South Africa; 2 Department of Mathematics and Actuarial Science, University of Stellenbosch, Stellenbosch, South Africa; University of Verona, ITALY

## Abstract

**Background:**

Emergency Departments serve as a main entry point for patients into hospitals, and the team, the core of which is formed by doctors and nurses needs to make sense of and respond to the constant flux of information. This requires sense-making, communication, and collaborative operational decision-making. The study’s main aim was to explore how collective, interprofessional sense-making occurs in the emergency department. Collective sense-making is deemed a precursor for adaptive capability, which, in turn, promotes coping in a dynamically changing environment.

**Method:**

Doctors and nurses working in five large state emergency departments in Cape Town, South Africa, were invited to participate. Using the SenseMaker® tool, a total of 84 stories were captured over eight weeks between June and August 2018. Doctors and nurses were equally represented. Once participants shared their stories, they self-analysed these stories within a specially designed framework. The stories and self-codified data were analysed separately. Each self-codified data point was plotted in R-studio and inspected for patterns, after which the patterns were further explored. The stories were analysed using content analysis. The SenseMaker® software allows switching between quantitative (signifier) and qualitative (descriptive story) data during interpretation, enabling more deeply nuanced analyses.

**Results:**

The results focused on four aspects of sense-making, namely views on the availability of information, the consequences of decisions (actions), assumptions regarding appropriate action, and preferred communication methods. There was a noticeable difference in what doctors and nurses felt would constitute appropriate action. The nurses were more likely to act according to rules and policies, whereas the doctors were more likely to act according to the situation. More than half of the doctors indicated that they found it best to communicate informally, whereas the nurses indicated that formal communication worked best for them.

**Conclusion:**

This study was the first to explore the ED’s interprofessional team’s adaptive capability to respond to situations from a sense-making perspective. We found an operational disconnect between doctors and nurses caused by asymmetric information, disjointed decision-making approaches, differences in habitual communication styles, and a lack of shared feedback loops. By cultivating their varied sense-making experiences into one integrated operational foundation with stronger feedback loops, interprofessional teams’ adaptive capability and operational effectiveness in Cape Town EDs can be improved.

## Introduction

Emergency departments (EDs) serve as entry points for patients into hospitals, providing care to unscheduled patients of every age, with a range of diagnoses and varying acuities. Uncertainty, interruptions, multiple–often conflicting–priorities, and gaps in information flow are inherent to ED work practices. These challenges are compounded by doctors and nurses having disparate task and workload dimensions yet remaining simultaneously independent and interdependent [1–3].

In many ways, the healthcare delivery system is designed to have different flows of information, with various acceptable methods for processing and responding to information or anomalies between professional roles. For example, in the ED, doctors and nurses work together in the same space and care for the same patients, sharing resources while adhering to different chains of command that feature contrasting policies, procedures, and rules. Other than the different flows of information, the inherent variability of the ED creates constant change, where available information may rapidly become dated, necessitating constant re-updating, efficient streams of information sharing, and ways of prioritising responses to information. These activities, concerned with figuring out what is happening and guiding the decisions (i.e., ‘what next’) can be grouped together as sense-making activities [4].

Because of its dynamic nature, the ED is a high-risk environment for operational failure. This term is used to refer to workarounds, duplication, process errors, failures in information flow, and other factors that contribute to disruptions, errors and insufficiencies in information, resources, or equipment [2]. Operational failure can also manifest as a wide-ranging set of small-scale issues rather than a single catastrophic event. However, sense-making, and subsequent adaptive capabilities can prevent operational failure and improve efficiencies in work processes. Adaptive capability situated at the operational level and embedded in team interactions is about anticipating and adjusting to real-time changes. It is the product of continuous sense-making, information-processing, interprofessional communication, real-time feedback, and the ability to modify resources and plans [5, 6].

Adaptive capability becomes vital in dynamic environments as no single individual can notice and respond to everything that is happening and changing. Instead, people select what is notice-worthy to them. This process is not straightforward, as sense-making depends on what people are conditioned to notice, influenced by perceptions regarding the professional role, experience and organisational rules [7].

Team members facing the same situation but with different conditioning will notice different signs or aspects of information or situations as important, with varied views on the next appropriate action. Also, gaps in the flow of information are filled with assumptions; when the assumptions are shared within a group with similar conditioning (e.g., single discipline), jointly held assumptions are likely to be strengthened, whereas perspectives could shift when the assumptions and information are shared in a heterogenous group [8].

In complex systems, the connections, interdependencies, and interactions are entangled, and it is the very entanglement that allows the system to perform [9, 10]. All interactions create patterns, observable at a systems level, but obscured when viewing parts in isolation. The complexity lens considers the dynamic interplay between the different parts as opposed to studying individual components, e.g., doctors or nurses in isolation. We undertook a study considering the ED as a complex system to explore the interprofessional ED team’s ability for sense-making when responding to operational challenges. Sense-making is deemed a precursor for adaptive capability, which, in turn, promotes coping in a dynamically changing environment.

Even though the SenseMaker® approach has been used extensively in organisations and is described in grey literature, there remains limited academic literature about the utility of SenseMaker® as a research tool [11]. This study adds knowledge about the research application and usefulness of the SenseMaker® tool.

## Methodology

This exploratory study investigated collective sense-making in five large, Cape Town-based state sector EDs. Of interest were interprofessional sense-making perspectives regarding the availability of information, the clarity of consequence of actions (decisions), and method of communication. The data used in this article were extracted from a larger study that included an observational study, semi-structured interviews, and the SenseMaker® study.

### Research setting and participants

With an estimated population of 3 740 026 people (2011 census), Cape Town is the largest city in the Western Cape Province of South Africa. This middle-income country has one of the highest health expenditures amongst middle-income countries that is contributed to the quadruple burden of disease–namely illnesses of poverty, non-communicable disease, HIV/AIDS, and injury [12]. The City of Cape Town has an unemployment rate of 23.7% and it is estimated that nearly 36% of households live below the poverty line. The unemployed and those living below the poverty line are likely to utilise district and regional EDs for emergency care needs. Traumatic injuries far exceed global averages and Cape Town has the highest murder rate among large South African cities, and the victims of crime often present at state EDs. Three out of four presentations to the ED are estimated to be due to complications of non-communicable disease [13]. A retrospective review of bed utilisation and occupancy in eight state hospitals in Cape Town revealed the average bed occupancy of EDs in the metropole fluctuated between 270–370% [14]. This implies that state EDs are severely overwhelmed, clinically with the presentation of a combination of trauma and non-communicable disease; and operational challenges such as crowding, flow, and staff shortages.

The study was conducted in regional and district hospitals in the Cape Town metropolitan area. Non-random purposive sampling was used to invite all categories of doctors and nurses employed at five large state EDs in the Cape Town metropolitan area to participate. Staff email addresses were provided by the operational managers and potential participants were invited via email.

### Inclusions

All doctors and nurses working in the five EDs, including contract staff.

### Exclusions

Patients, visitors, prehospital medical services, and non-clinical functions, e.g., administrators, porters, and security staff.

### The SenseMaker® method

SenseMaker® is a suite of software tools developed by the Cynefin Company. The tool extensively employs visualisation to uncover patterns and relationships in the data. In SenseMaker®, the underlying theory to explore is selected before survey design; in this case, the theory used was the sense-making process, as described by Weick, Sutcliffe and Obstfeld [7].

SenseMaker® surveys start with an elicitation question, prompting participants to tell a short descriptive story. The micro-narratives (short descriptive stories) provide more sense-making context than structured narratives [11]. For this study the prompting question presented participants with an interruption, the nature of which they could choose and disclose. After sharing their story, participants are led through a series of specialised questions, called signifiers, that allow them to self-analyse their story within the pre-designed framework. The short descriptive story and signifier data are captured in a common database that visually displays data for further analysis. The software allows switching between quantitative (signifier) and qualitative (descriptive story) data during interpretation, enabling more deeply nuanced analyses.

There is no required sample size for SenseMaker® studies, but capturing more stories means that the emerging patterns will be stronger [15]. The only precondition for the study was to proportionally represent both doctor and nurses.

### Data collection

We used a mix of web-based and paper-based surveys to collect data since some participants did not have email or web access. Data collection took place over 8 weeks in June—August 2018.

The plan was to only use the web-based application, but we reverted to paper-surveys as some staff members had no email or web access. Page one of the survey contained information regarding the study and the consent form. On the web-based applications participants were required to give consent before they could proceed to the rest of the survey. Page two contained demonstrations of how to interpret their story using the signifiers. All SenseMaker® data was stored in a secure third-party database, while paper copies were collected and kept at an off-site location.

### Data analysis

Data were captured into SenseMaker® Collector software, from where signifier data were uploaded into R Studio version 1.1.463 (2009 to 2017) for a statistical exploration of the quantitative signifier data. The standard formats of the triads and dyads, each yield characteristic types of data that can be visualised in R Studio on different ways, e.g., the triads were plotted on a ternary coordinate system with three components using a R-package specifically for ternary plots, namely ggtern (version 3.0.0.1). Density curves of dyads are plotted on the normal cartesian coordinate system using ggplot 2 (version 3.1.0). Another package for visualisation, ggridges calculates density estimates and then creates partially overlapping plot lines using ridgeline visualisation (version 0.1.5).

The steps followed for analysis of the signifiers included plotting each signifier, and inspecting it for patterns, after which patterns were explored by filtering it with demographic data e.g., professional role. The descriptive stories were analysed using content analysis as described by Erlingsson and Brysiewicz [16]. The completed story set was only read after the SenseMaker® Analysis was done.

### Ethical approval

Ethical approval was obtained from the University of Cape Town’s Health Research Ethics Committee (HREC 487/2017). Thereafter, permission to conduct both phases of the study at the selected EDs was obtained from the Western Cape Department of Health.

Participants provided informed consent, and they could withdraw from the study at any point until data analysis commenced. The SenseMaker® tool scrambles meta-data to reduce the possibility of participant or location identification. Participation was seen as a low-risk activity, and counselling services were available should the survey cause distress to participants.

### Trustworthiness

The following measures were taken to ensure trustworthiness:

The study was not aimed at being generalisable, nor can it be replicated with the same results. Occurrences in complex systems are not repeatable, and even though some themes may be generalisable to the process of sense-making, the study was not designed to build predictive models of sense-making [17, 18].

To ensure credibility, the findings discussed in this article are part of a larger study, and phase one of the full study included prolonged and regular engagement with the EDs over five months. Documentary resources including policies and procedures where perused and compared across roles and departments. During this phase member checking was done by informal, unstructured interviews. Daily processes such as handovers and other routines were observed for a total of 53 hours.

To ensure dependability, the study was designed with overlapping methods; field notes and a reflexive journal were kept [18, 19]. Moreover, a narrative, critical account of self-dialogue was kept as part of a reflexive journal that provided information on the logic of the process. This made conclusions traceable and increased rigour. Confirmability was achieved in various ways; firstly, by participants’ self-interpretation of their own stories and, secondly, because SenseMaker® allows for multiple perspectives of a similar experience. SenseMaker® creates distance between the researcher and interpretation, as participants’ self-interpretation of the narratives is displayed as quantitative data before any researcher-manipulation of the data occurs. To ensure fairness the sampling strategy included the precondition of proportional representation of both roles. This meant switching from the original plan to only use web-based surveys to using a hybrid method of web-based and paper-based surveys.

## Results

For the SenseMaker® part of the study, 84 stories were collected; doctors and nurses were equally represented (Table 1). All categories of doctors and nurses working in the five selected EDs were represented, with the senior roles–specialist emergency physician and professional nurse–having the best representation.

**Table 1:** Breakdown of participants within doctor and nurse categories.

Category	Description	Participants
Emergency Physician	Medical Specialist in Emergency Medicine.	24
Medical Officer	Doctor that completed medical degree and internship.	18
Professional Nurse	4-year degree or Diploma nurse. Independent practice	25
Enrolled Nurse	Educated to practise-based nursing. Supervised practice.	14
Nurse Assistant	Educated to provide basic nursing care. Supervised practice.	3
Total		**84**

The results focused on four aspects of sense-making, namely views on the availability of information, the consequences of decisions (actions), assumptions regarding appropriate action, and preferred communication methods. These processes enable (or are required for) adaptive capability.

### Perspectives regarding the availability of information and clarity on the consequences of decisions

**Fig 1:**
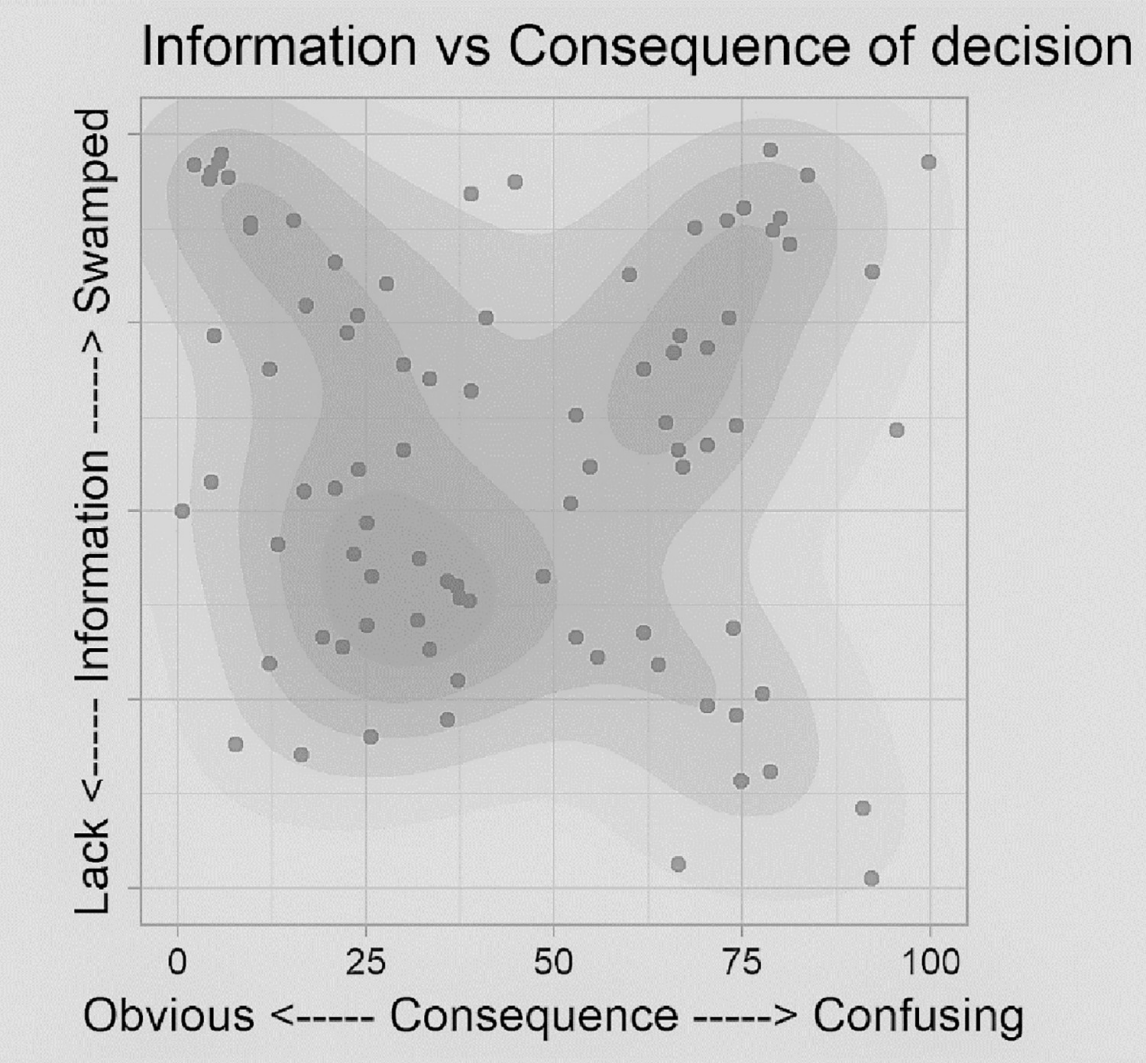
Connection between information flow and the consequences of decision-making.

Fig 1 combines the responses to two survey questions presented in a dyad format. Dyads have two extremes on a linear scale, with one extreme indicating the underlying construct to be completely absent and the other extreme presenting the construct in excess. This may appear similar to other commonly used linear scales, but the difference is that the preferred state is somewhere between the two extremes [11].

The first dyad asked: *In your story*, *the consequence of the decisions that had to be made in the ED was…* and the extremes were *blindingly obvious* versus *confusing and uncertain*. Responses are shown on the x-axis. The second dyad (shown on the y-axis) asked: *The availability of the required information was…* with the extremes being *too little*, *too late*, *too hard to find* versus *too much*, *too early*, *swamped with it*.

The three clusters identified in Fig 1 are:

Top left: Feeling swamped with information, yet the consequence of the decision is obvious

Top right: Feeling swamped with information, yet confused about the consequence

Bottom left: Enough information and obvious consequence of the decision

We consulted the narratives to understand the clusters and found two patterns at the swamped extreme of information. The top left corner shows those who viewed the outcome of their decision as obvious, and, when accessing their stories, they tended to offer solutions to the predicament in their stories, despite not being prompted to do so.

‘*We need management to take responsibility*. *Somebody needs to stand up and do more than just another meeting*.’ Doctor

The top right corner shows those who felt swamped with information, where the consequence of the decision was unclear, and they shared stories of dealing with conflict, multiple high-density tasks and feeling isolated.

‘*We are always interrupted and busy and sometimes you go home*, *and you do not know if you did everything*. *All that you can do is to keep going’* Nurse

The ideas expressed by participants at both extremes are contrasted in Table 2.

**Table 2:** Stories told at each extreme on the clarity of the consequence of the decision.

Blindingly obvious	Confusion and uncertainty
Use set ratios for staffing (Doctor)	Alone in the resuscitation room, with a nurse not understanding the seriousness of the condition (Doctor)
Involve inpatient teams to do their bit (Doctor and Nurse)	Doctors not considering nurses’ inputs (Nurse)
Solve the hospital issues that manifest in the ED (Doctor)	No orientation received on the first day (Doctor)
Psychiatric patients should go straight to the relevant ward (Nurse)	Dealing with multiple serious patients at once with no help (Doctor)

### Assumptions regarding appropriate action

**Fig 2:**
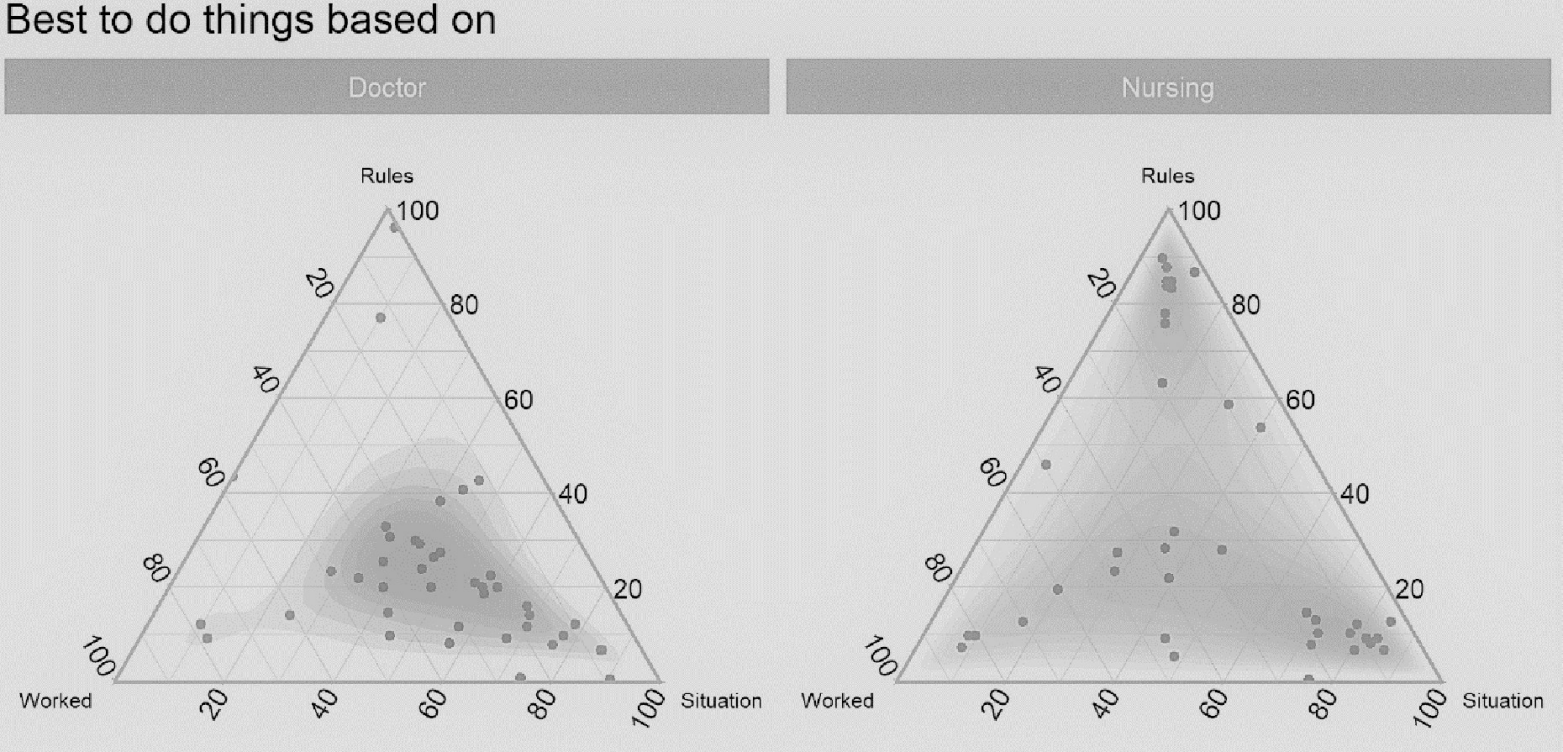
Doctors’ and nurses’ perceptions of appropriate action.

Fig 2 represents a triad; triads are triangular grids with labelled variables forming the corners. The interior of a triad represents the relative proportions of the three corner variables. The participant places a dot anywhere inside the triangle to demonstrate how the three variables at the corners trade off against each other. In Fig 2, we show the differences in perceptions among professional roles.

Storytellers who placed a dot in the middle of the triad indicated the three corners are weighted equally, implying that the best course of action was contextual. Otherwise, there was a noticeable difference in what doctors and nurses felt would constitute appropriate action. The nurses (right-side triad) were divided, with clustering at ‘following the rules and policies’ or ‘according to the situation’, whereas doctors were more likely to act ‘according to the situation’.

The following narrative enforces the quantitative data by demonstrating the tension between a doctor wanting to manage pain ‘according to the situation’ and a nurse wanting to ‘follow the rules and policies’.

*‘It was for a nurse issue*, *I prescribed morphine for a patient that was being nursed in a chair*. *They can’t nurse a patient that had morphine in a chair*, *the patient must be in a trolley*. *But there are no trolleys*. *So*, *what do you do*? *Manage the pain or ignore the pain*? *You can’t tell me not to write it up*. *How about I write it up and you tell me that you refuse to give it and write it in your chart*.*’* Doctor

### Preferred communication methods

**Fig 3:**
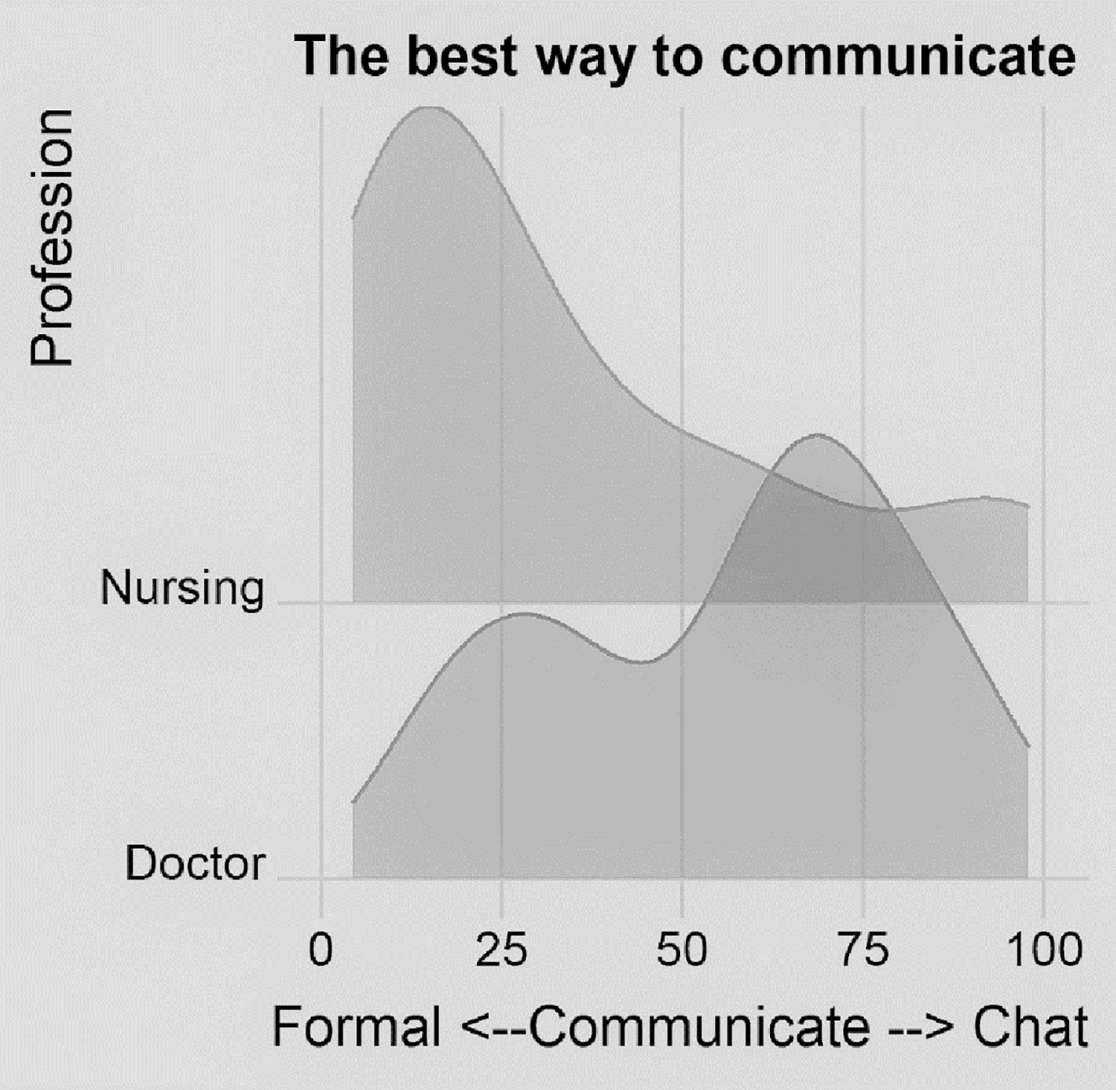
In your story, the best way to communicate was.

The dyad dealing with communication asked: *in your story*, *the best way to communicate was formal channels versus informal conversations*. The responses were contrasted based on the participants’ professional roles. Fig 3 shows the densities of the responses provided by the different professional roles. The height of the curve above the baseline represents the density of the responses, i.e., high peaks indicate many responses at close to that point in the scale.

More than half of the doctors indicated they found it best to communicate informally, whereas nurses indicated that formal communication worked best for them.

## Discussion

The findings present a picture of disjointed information-processing, weak interprofessional communication, and a lack of feedback channels that impedes effective sense-making and subsequent adaptiveness to unfolding situations. The interactions and interdependencies appear weak, and the operational disconnect between and within the interprofessional team is seen in all four aspects of sense-making, namely views on information, the consequence of the decisions (actions), assumptions regarding appropriate action (Figs 1 and 2), and preferred communication methods (Fig 3). The operational disconnect hampers individuals’ adaptive capability in the ED and may lead to a multitude of failures in this context.

A substantial number of participants indicated they felt overwhelmed with the information flow (Fig 1), and some were also confused about the potential consequence of any next steps. When people experience an information overload, they become blinded to additional information, ignoring subtle cues. They also lose their ability to patch together cues that, if picked up earlier, may have elicited a proactive response as opposed to responding to a crisis [20–22]. Due to the dynamic nature and constant flux, the ED is already a high-risk setting for operational failure, and it has been found that subtle changes are more likely to be selectively ignored in environments with information overload and fragmented communication [20]. When team members feel overwhelmed by the flow of information, cannot piece together the next steps and are unable to make sense of the situation, it threatens adaptive capability, compounding the risk of operational (and possibly clinical) failure.

The greater disconnect is seen in how everyday situations are dealt with (Fig 2). Some (mostly doctors) treat every situation as novel, regardless of the frequency of occurrence, while the rest (mostly nurses) blindly follow the rules, regardless of the rules’ appropriateness. Rigid adherence to rules regardless of the situation constrains adaptive capability [20]. The combination of feeling overwhelmed with information (Fig 1) and rigid adherence to rules (Fig 2) implies that potentially crucial information may go unnoticed. However, continuously acting according to the situation (i.e., treating each situation as if it is novel) implies a lack of shared learning and feedback loops. Neither is ideal, and pliancy is required between blindly following rules and treating each situation as novel. This pliancy can be achieved by improving sense-making via strengthened feedback loops and communication structures, not only between the professions, but also within the professions.

The importance of communication during sense-making is illustrated in Table 2. Those who felt overwhelmed by information and uncertain of the consequence of next steps shared stories that dealt with communication. Conversely, those who felt overwhelmed with the obvious consequences of decisions were likely to offer solutions that did not fall under their direct control.

Fig 3 suggests that doctors and nurses prefer different communication methods. Other studies have suggested that nurses use indirect methods to communicate or may choose not to speak up even in life-threatening situations. Our study implies another possibility: even when nurses speak up, they might not be ‘heard’ by the doctors due to the communication methods used, and doctors and nurses may be speaking past each other. Different communication methods may result in vital knowledge remaining trapped within a discipline. This requires further research, as breakdowns in communication are widely described as causal to systems failure and medical error [10].

A correlation has been found between operational failure, job performance and level of burnout, and reducing the risk of operational failure might improve how ED staff experience their work environment [2]. Applying simplistic linear solutions to complex systemic problems risks interrelated factors being overlooked, resulting in deeper underlying causes remaining unresolved [7]. It is imperative to move beyond traditional notions of parallel discipline-specific sense-making–where doctors and nurses function as segregated groups–towards an ED team identity with a ‘central’ ED information exchange platform, continuously sharing knowledge across interprofessional boundaries.

This study contributes empirical knowledge as most studies done in EDs are focused one discipline or on a singled-out process condition. Theoretically the study adds insights into how sense-making occurs within a dynamic interprofessional setting and the findings/patterns emerged from the five EDs that participated in the study. Methodologically the study contributes to the growing body of work on SenseMaker®. The SenseMaker® tool provides unique opportunity to explore the human dynamics within complex systems [11]. Advantages of using the method includes the ability to merge qualitative and quantitative data for nuanced analysis, the distance created between the researcher and interpretation of data to reduce bias, and the broad elicitation question permits a wide range of stories and interpretations by the participants. Limitations of the SenseMaker® tool included the initial investment to become proficient in design and analysis of SenseMaker® studies and some participants struggled with the concept of self-codifying their narratives.

### Limitations

In this study, we considered doctors and nurses working in the ED and excluded other groups, such as administrators. However, as most operational decisions are made by doctors and nurses, this should not present a major limitation.

We also excluded demographic variables, e.g., race and gender. Including these variables would have provided more individual perspectives, potentially distracting from the purpose of the study, which was centred on the interplay between the professions.

## Conclusion

This study was the first to explore the ED’s interprofessional team’s adaptive capability to respond to situations from a sense-making perspective. The ED is a dynamic setting with high levels of variability, and functioning relies on the strength of interactions and interdependencies in the complex system. By cultivating their varied sense-making experiences into one integrated operational foundation with strong feedback loops, interprofessional teams’ adaptive capability and operational effectiveness in Cape Town EDs can be improved.

## Recommendation

Adaptive capability is situated at the operational level and can be amplified by improving interprofessional sense-making. Doctors’ and nurses’ different conditioning, perspectives and priorities present an untapped sense-making opportunity that should be exploited. We recommend that the EDs adopt an integrated approach for dealing with information, communication, and feedback loops. This means integrating formal operational structures, e.g., one set of shared operational rules and policies. Special attention should also be paid to promote interprofessional communication practices that are frequent, timely, accurate, and sent via focused channels. Redesigning communication systems to allow reciprocal horizontal relationships would also improve knowledge management [3].

## Supporting information

S1 Appendix Web-based survey. (DOCX)

S2 Appendix Single signifier results. (DOCX)
