# Supplementary material for: Interprofessional sense-making in the emergency department: A SenseMaker study
Source: PLoS One. 2023 Mar 9;18(3):e0282307. doi: 10.1371/journal.pone.0282307 (PMC9997966; doi:10.1371/journal.pone.0282307)

Web-based survey

The survey was captured in the SenseMaker® patented software, but data is belongs to the researcher.


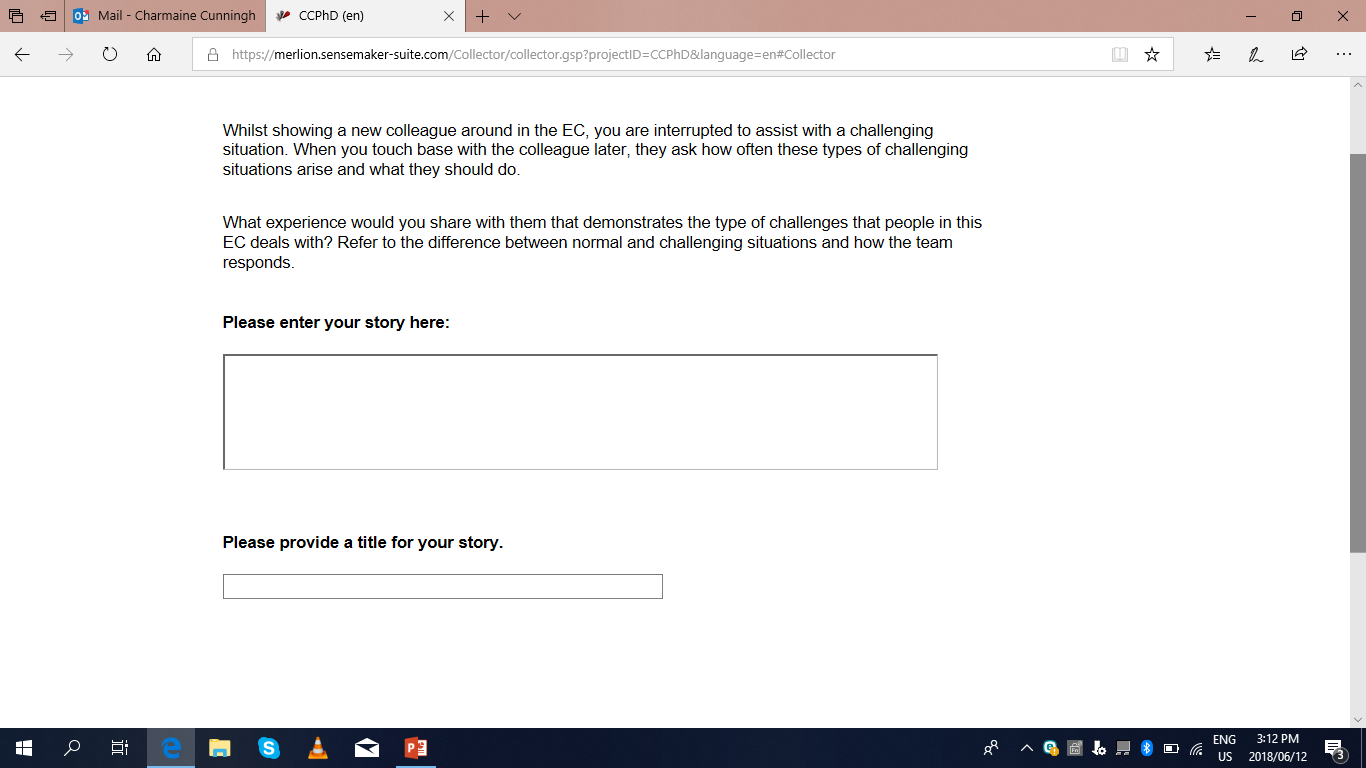


|  |
| --- |


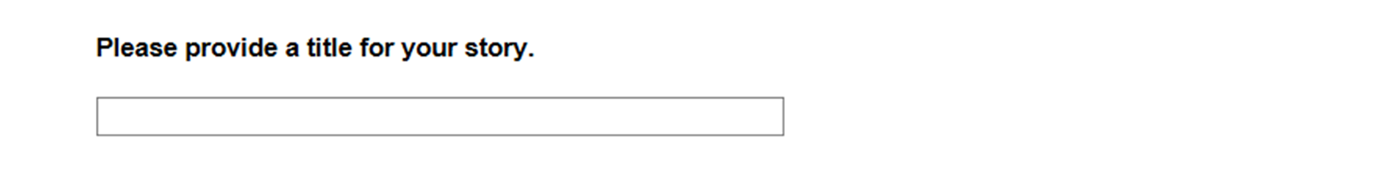


The following questions will use a series of triangles with words at each corner. Please place a dot closest to the position that you feel best represents your story. The closer you place the dot to the word (s), the closer the match with your story. Placing the dot in the middle of the triangle, equal distances from the corners, indicates that all three elements are equally represented in the story. Thinking about the story that you just shared, please answer the following questions


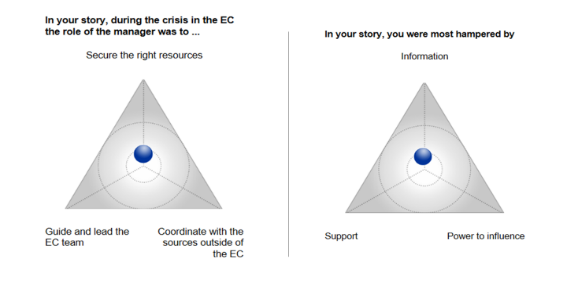


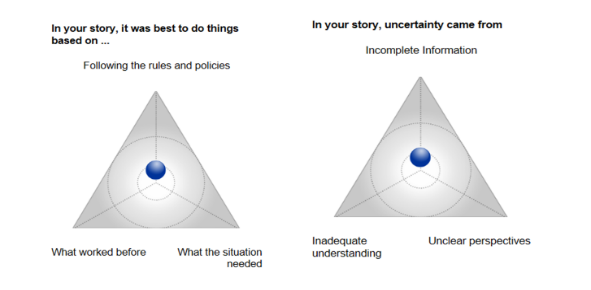


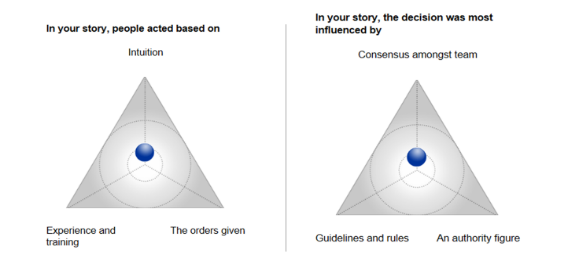


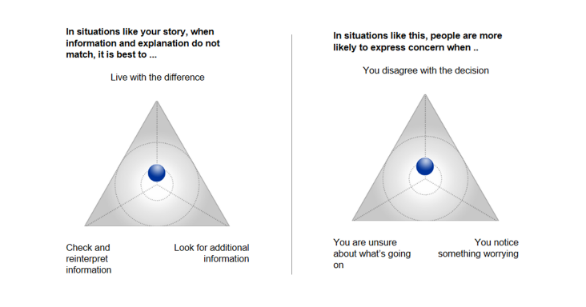


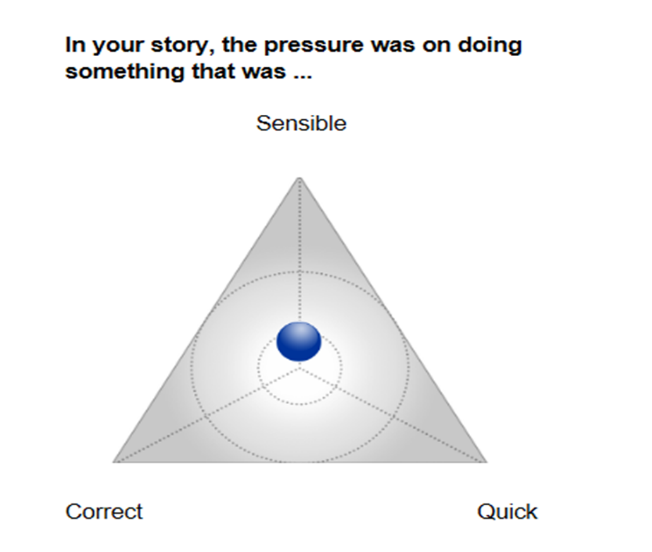


The following questions will use a series of bars with a word at each end. Please move the dot to the position that you feel best represents your story. The closer you move your dot to the words, the more it matches with your story. Moving the dot to the middle of the bar indicates that both elements are equally represented in the story.


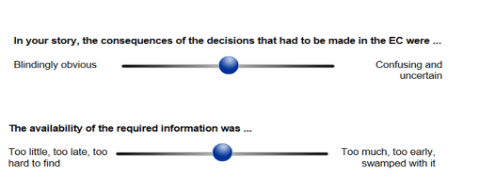


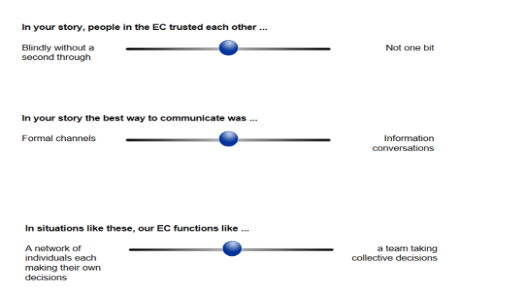


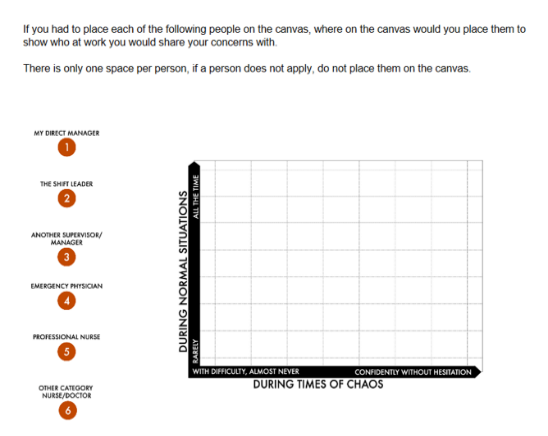


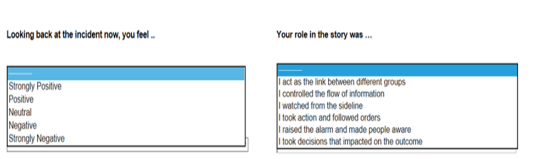


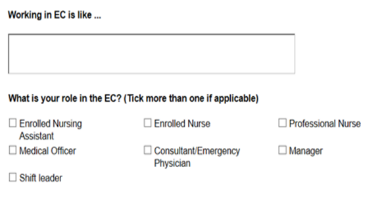


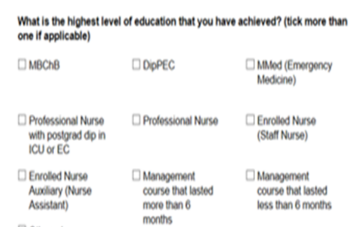


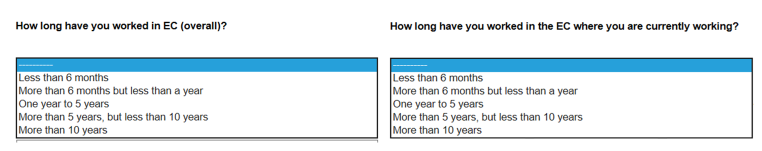

Supplement: S1 Appendix — (DOCX) [file pone.0282307.s001.docx]
