# Supplementary figures and images for: Interprofessional sense-making in the emergency department: A SenseMaker study
Source: PLoS One. 2023 Mar 9;18(3):e0282307. doi: 10.1371/journal.pone.0282307 (PMC9997966; doi:10.1371/journal.pone.0282307)

Single signifier results


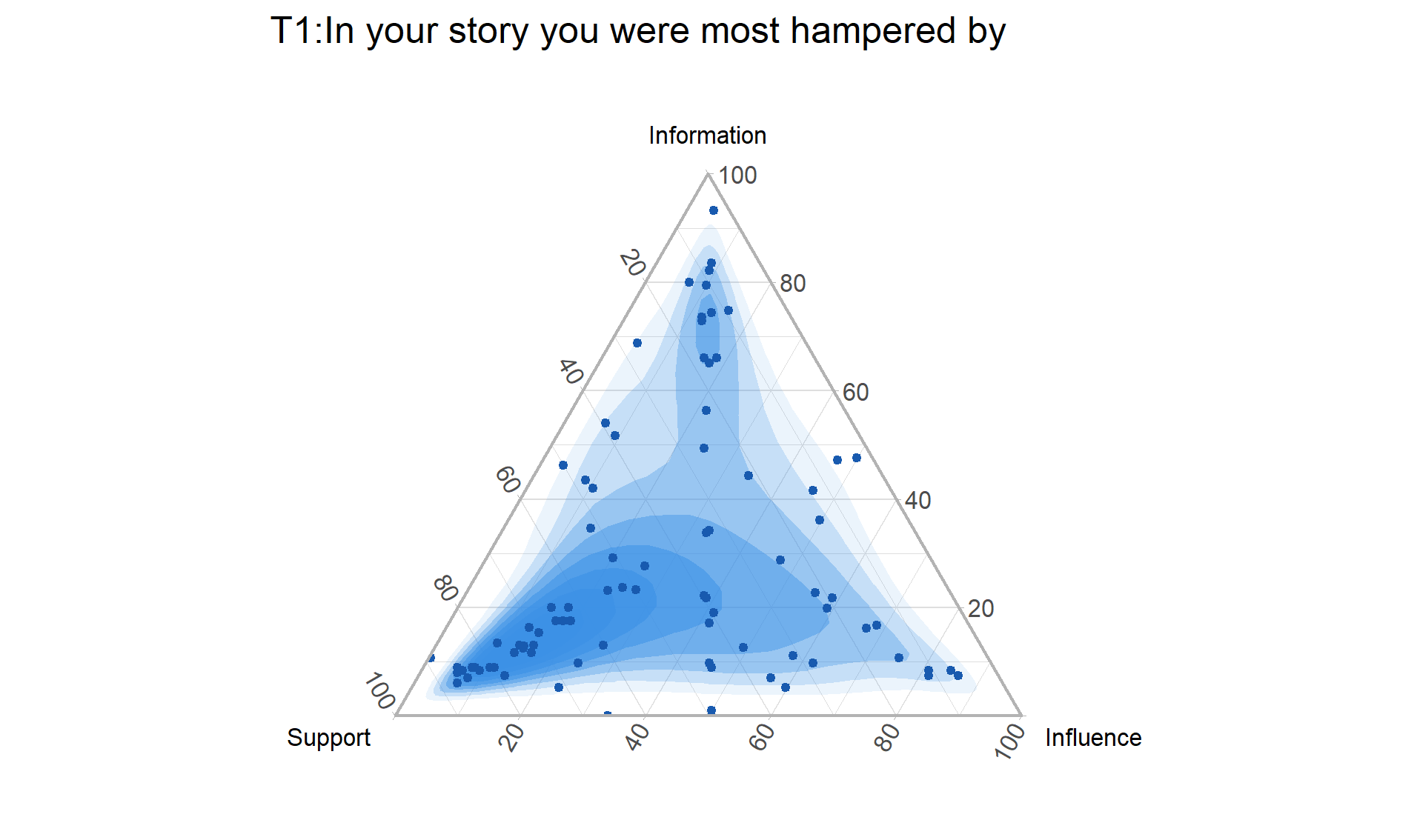


**
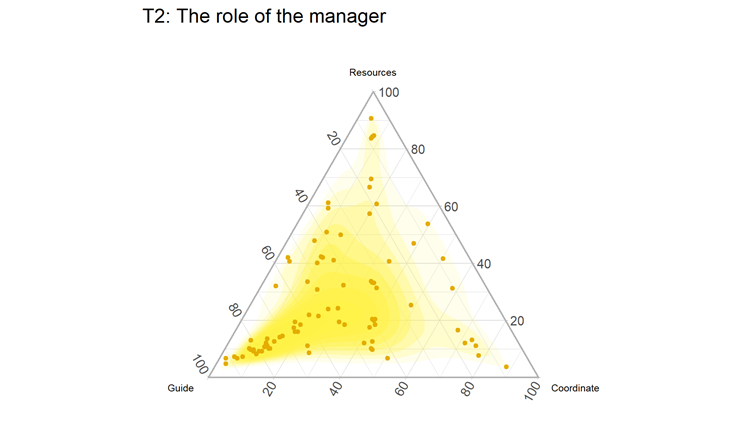
**


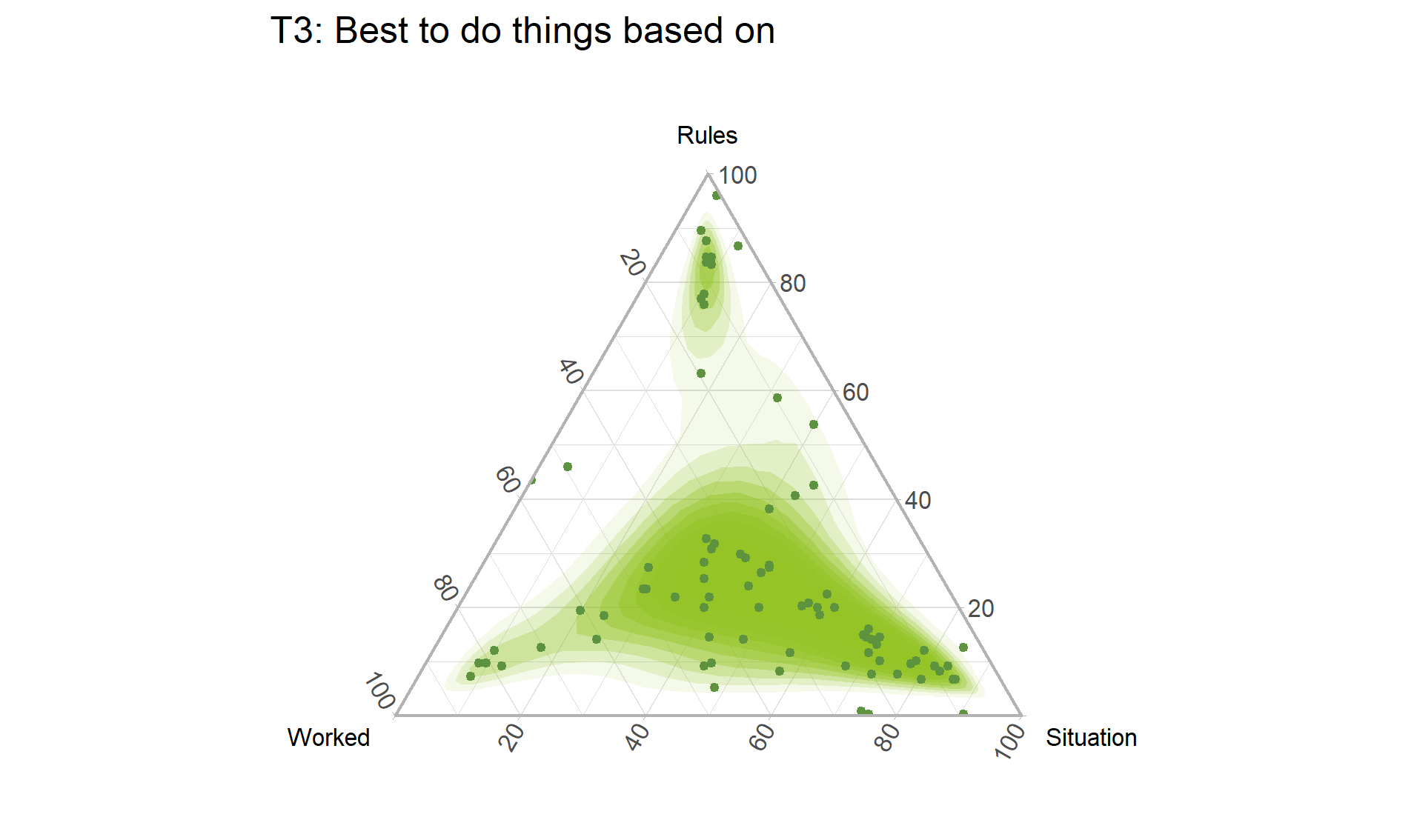


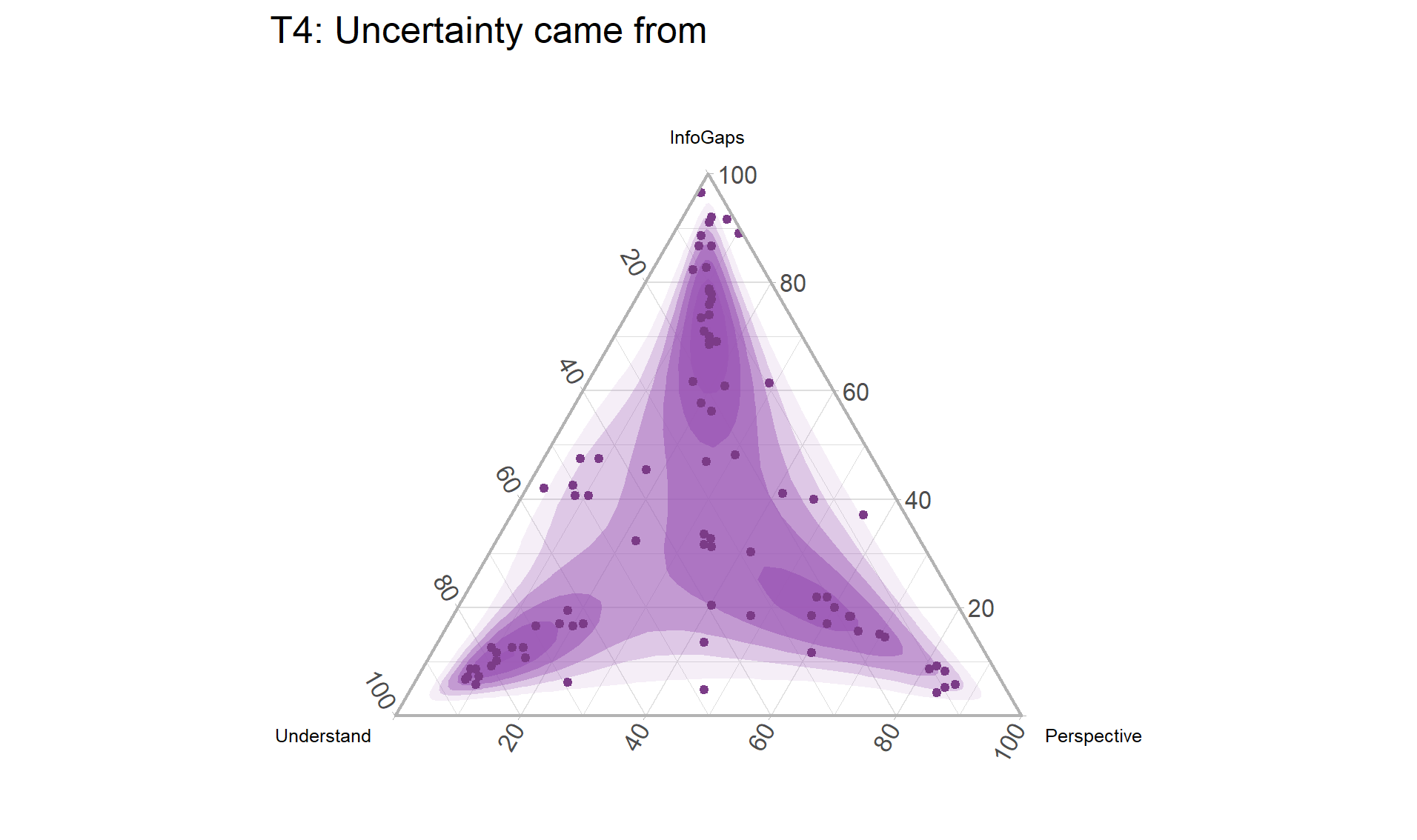


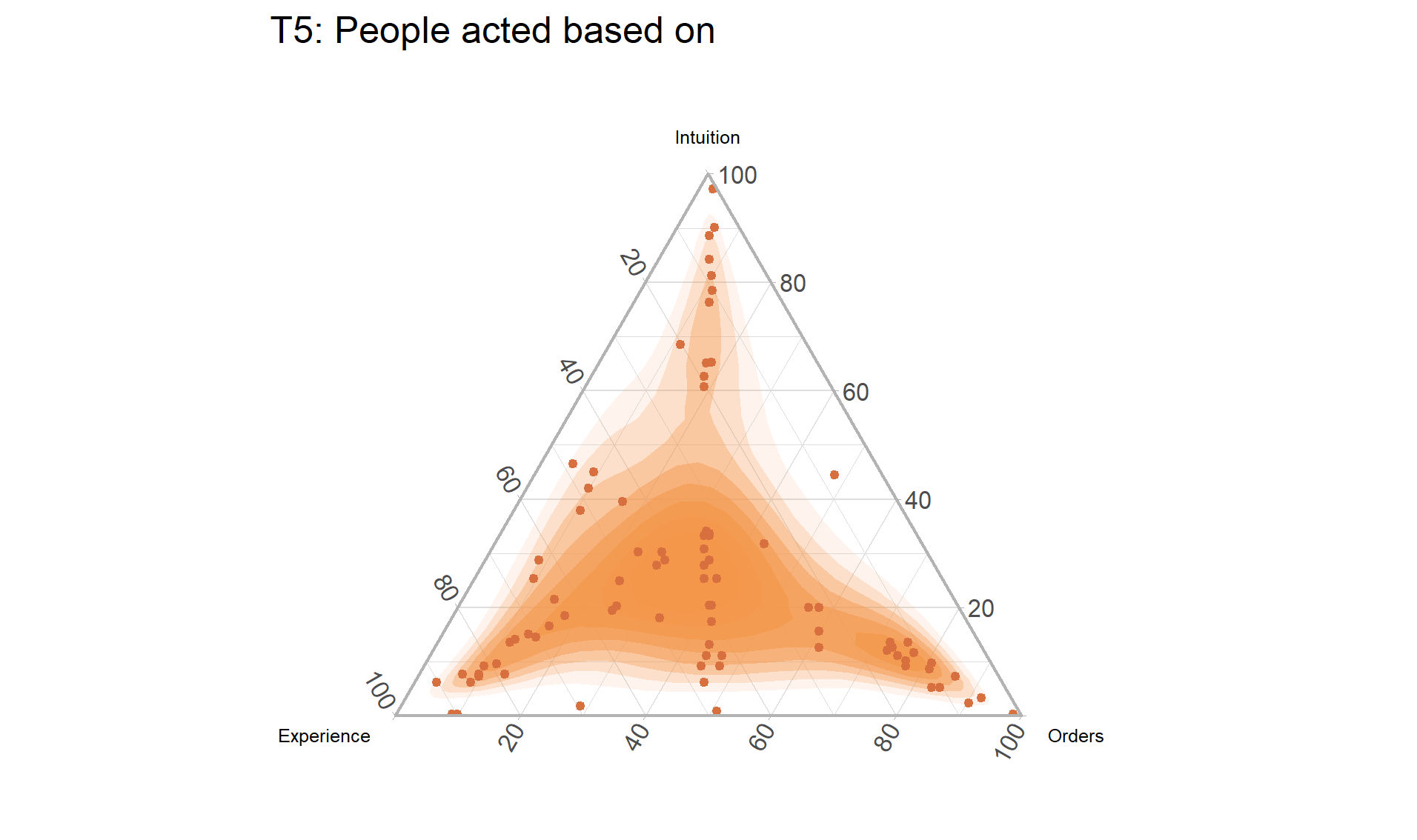


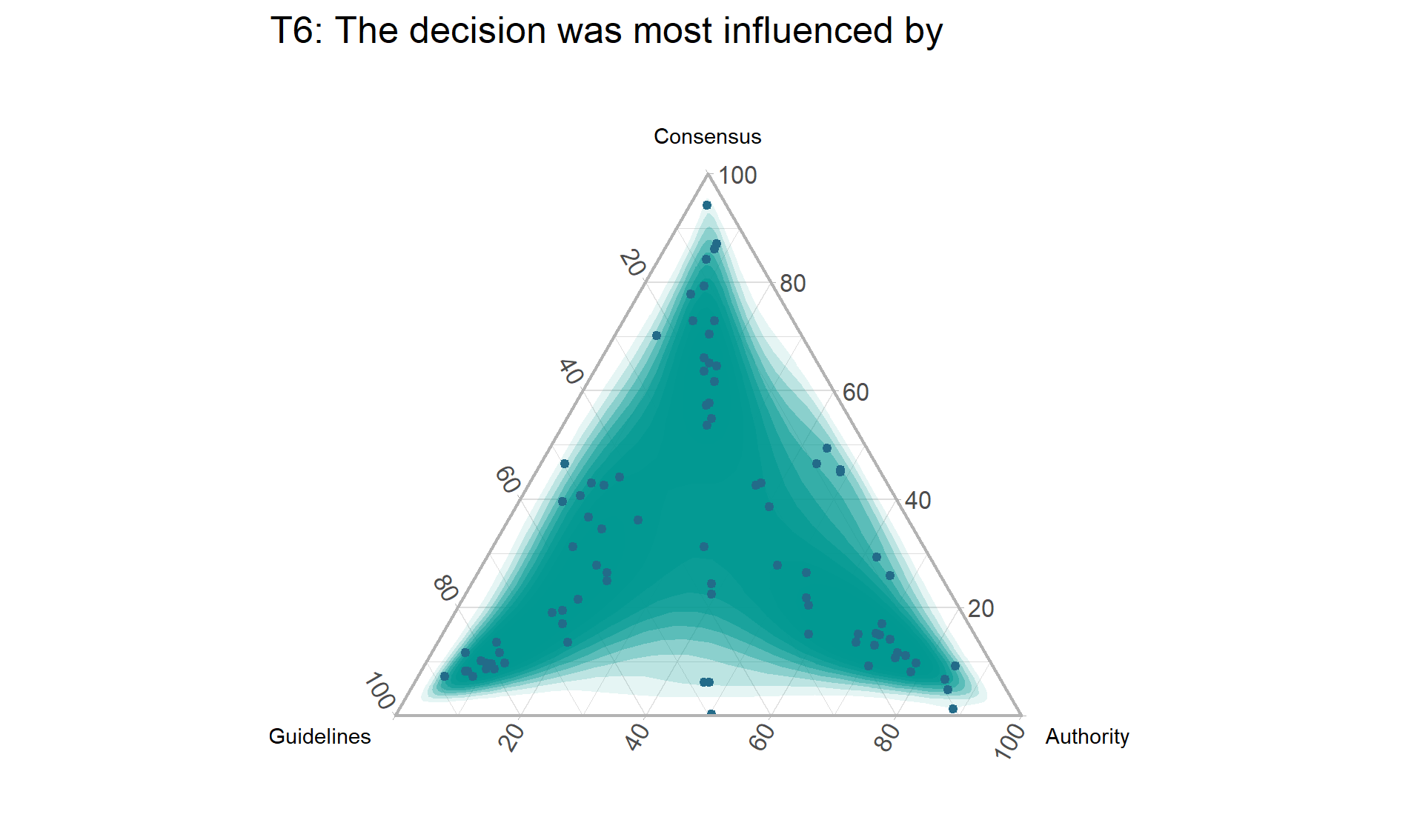


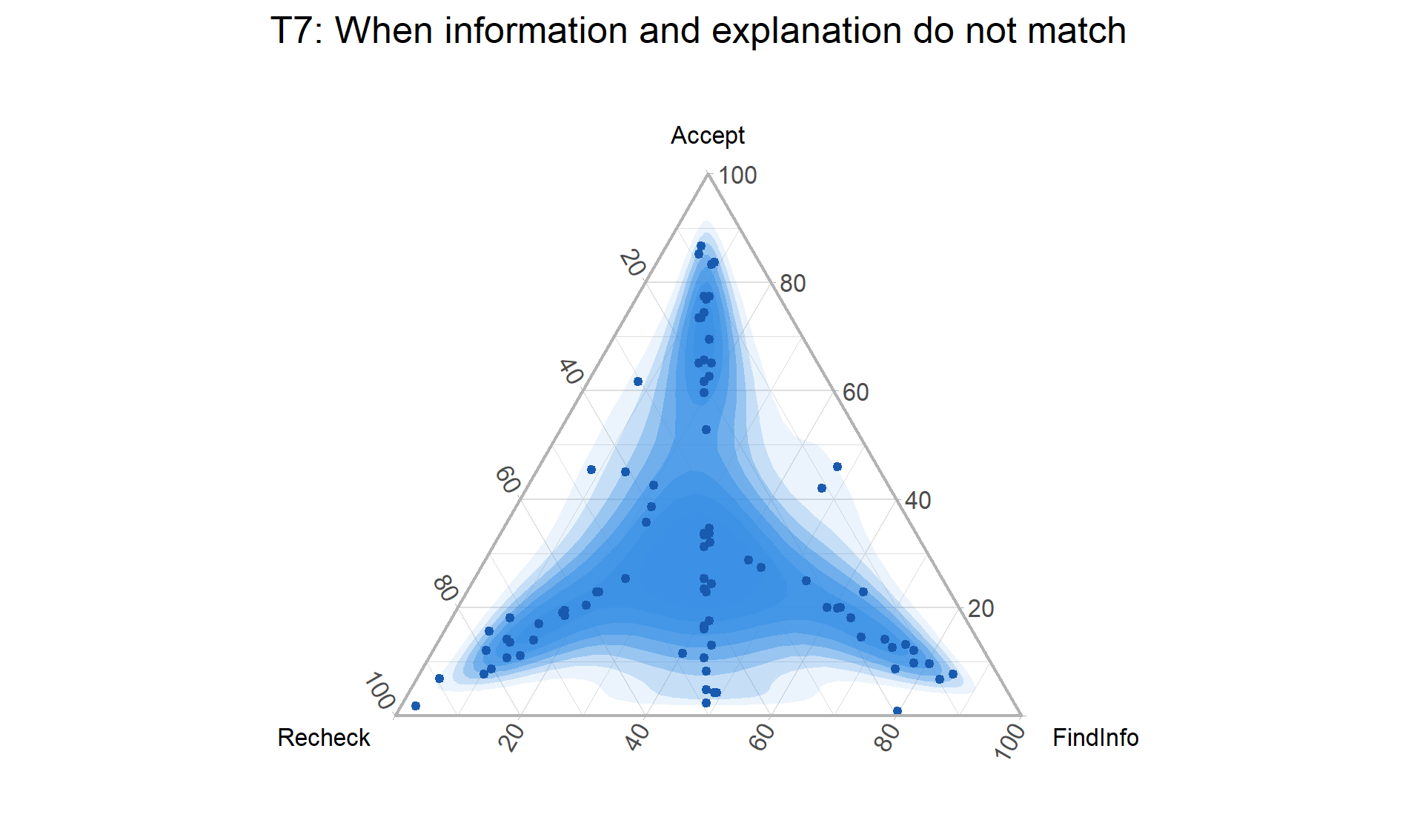


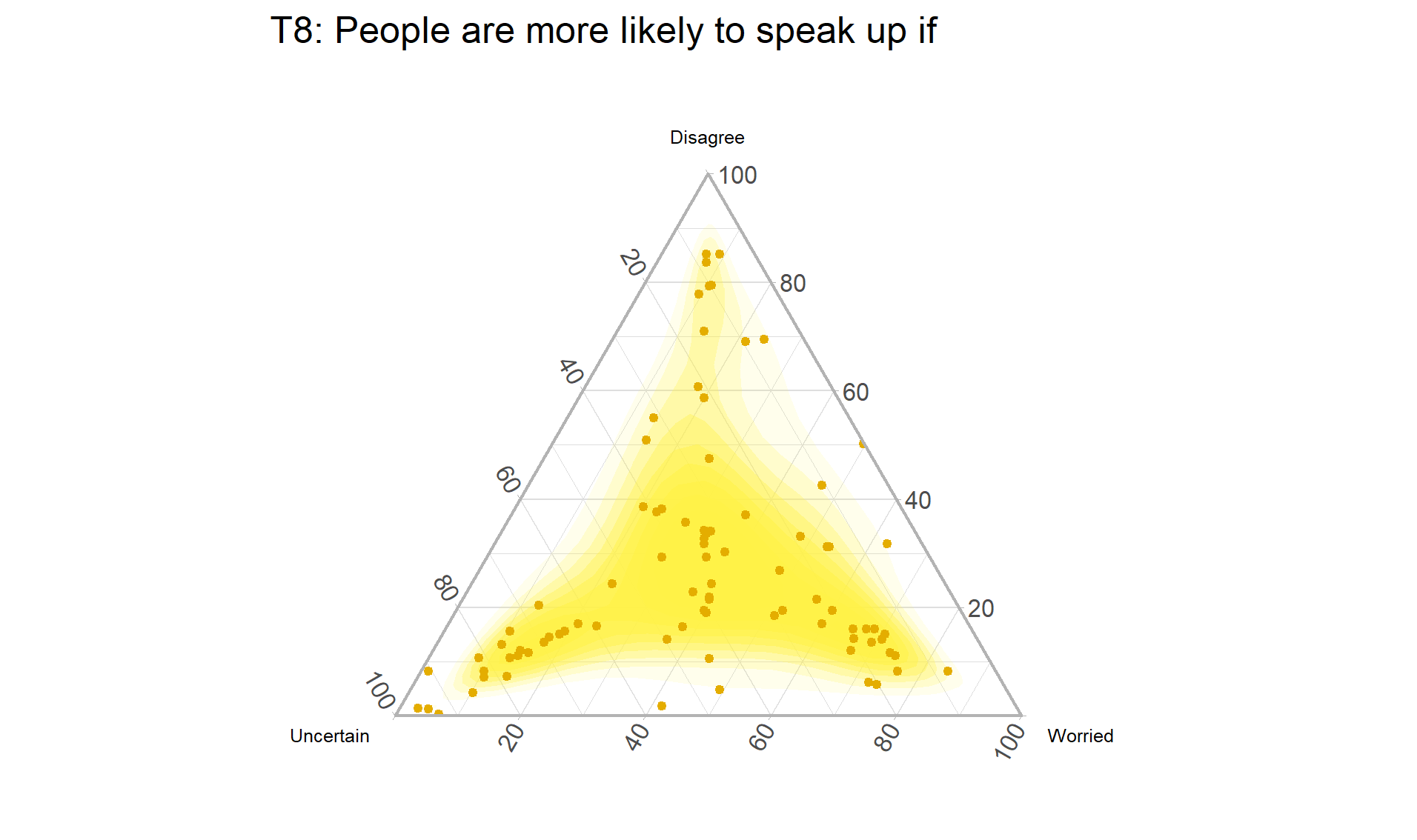


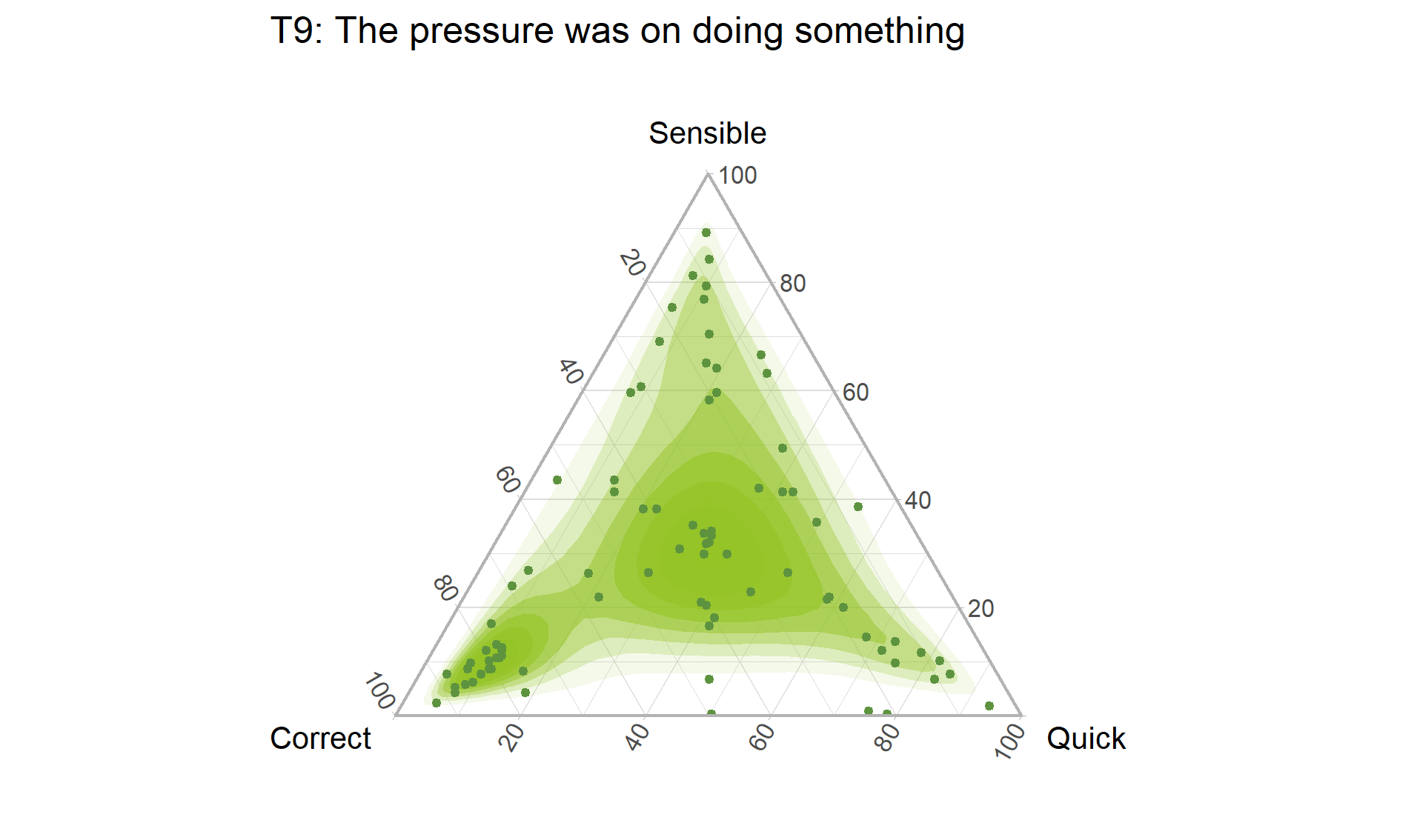


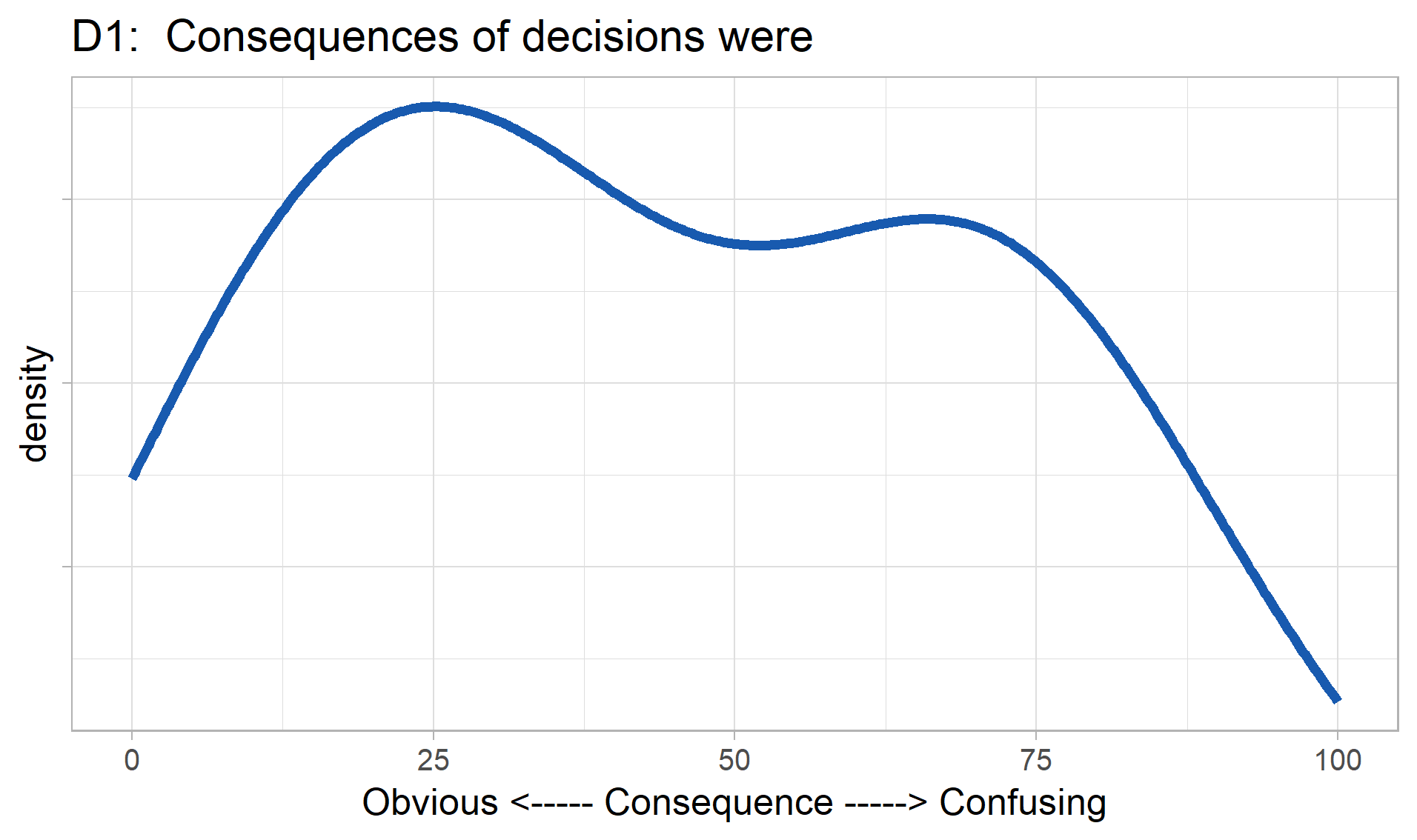


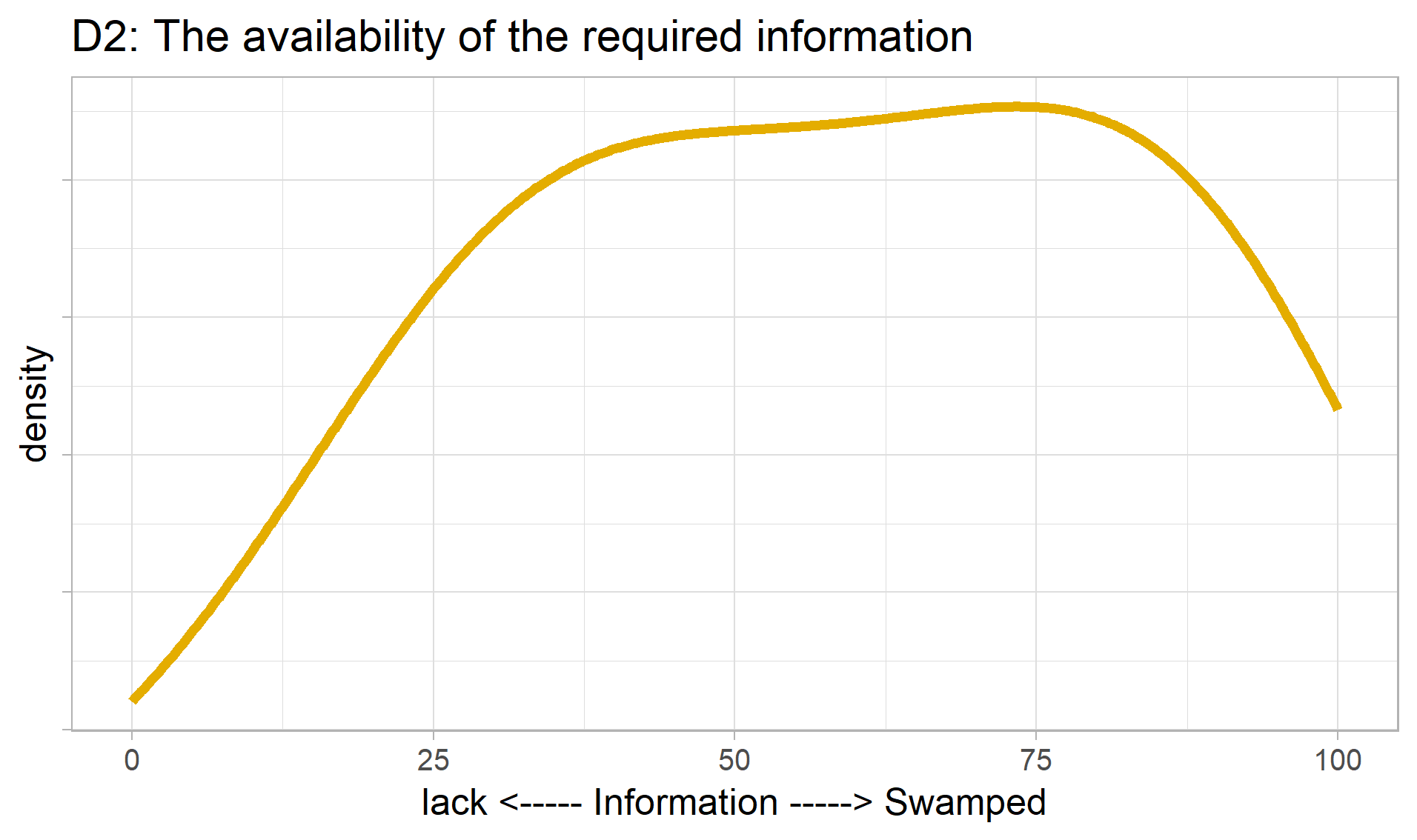


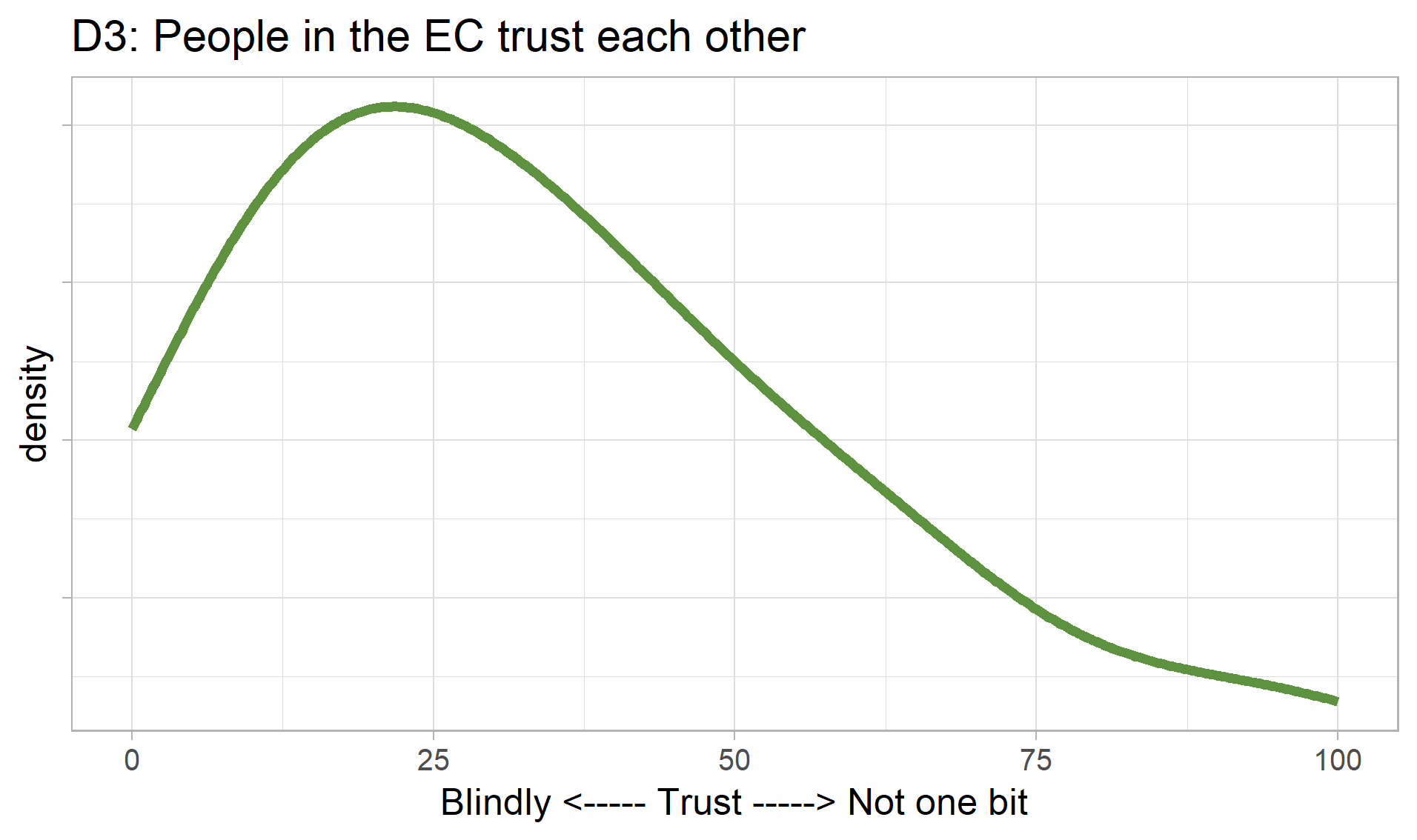


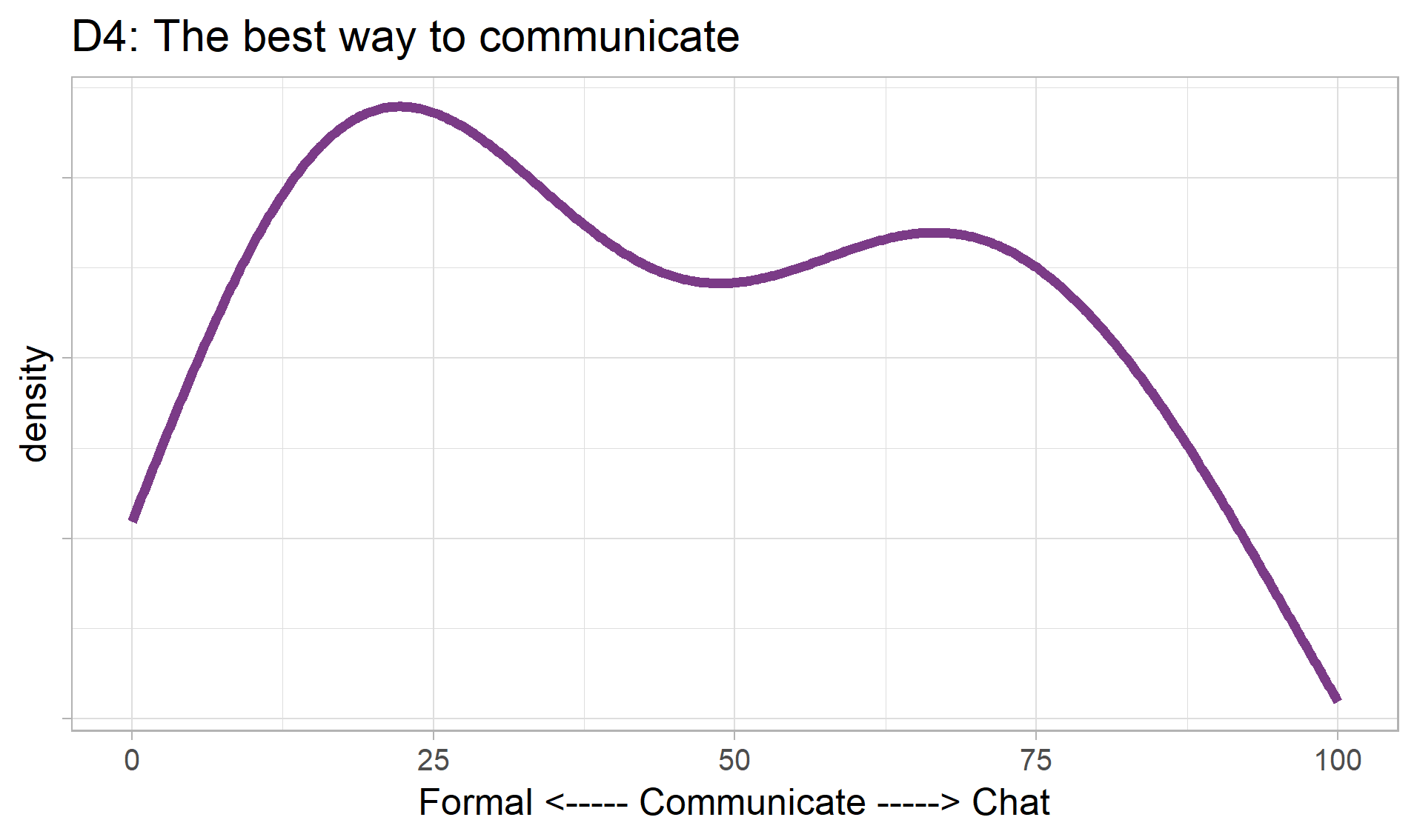


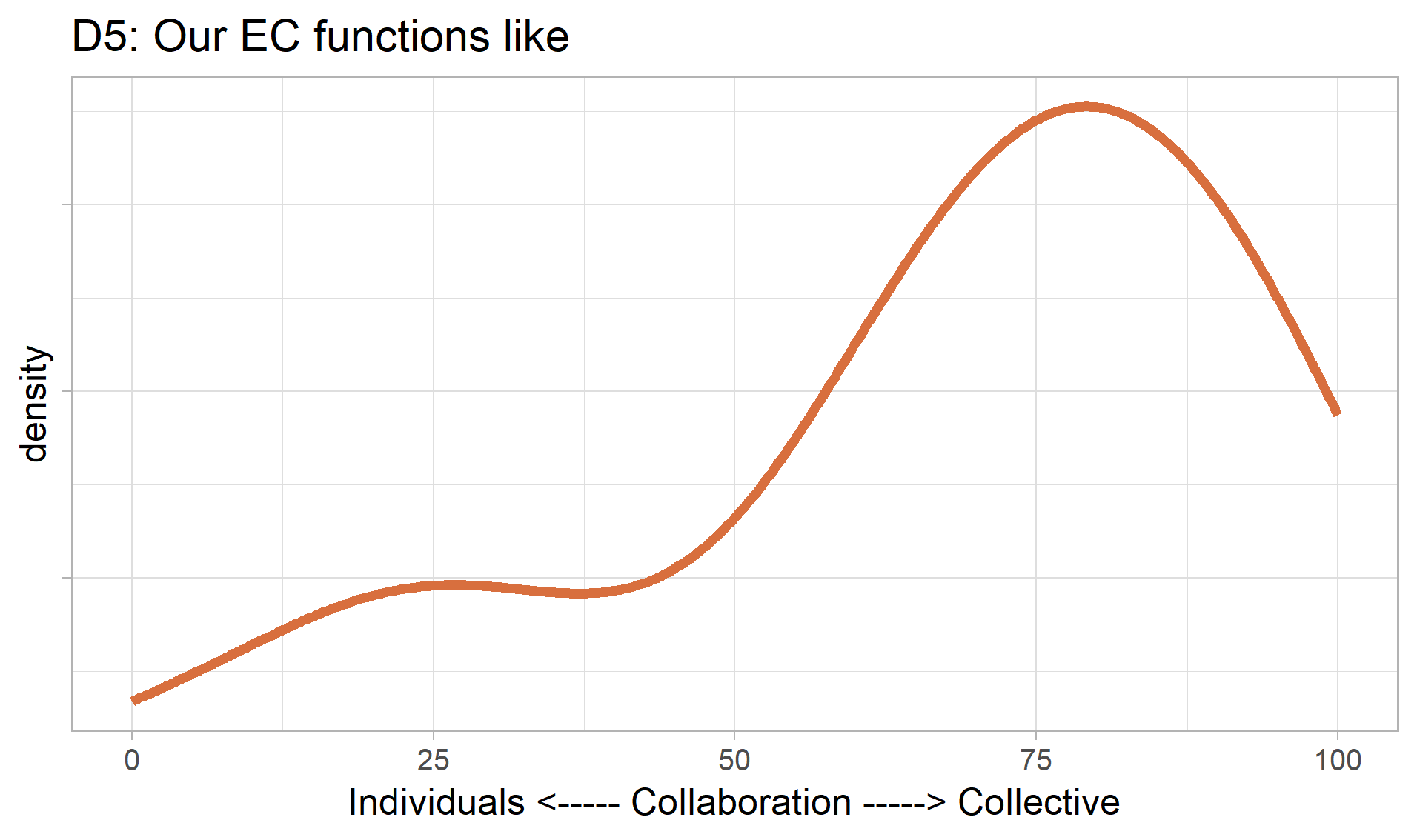


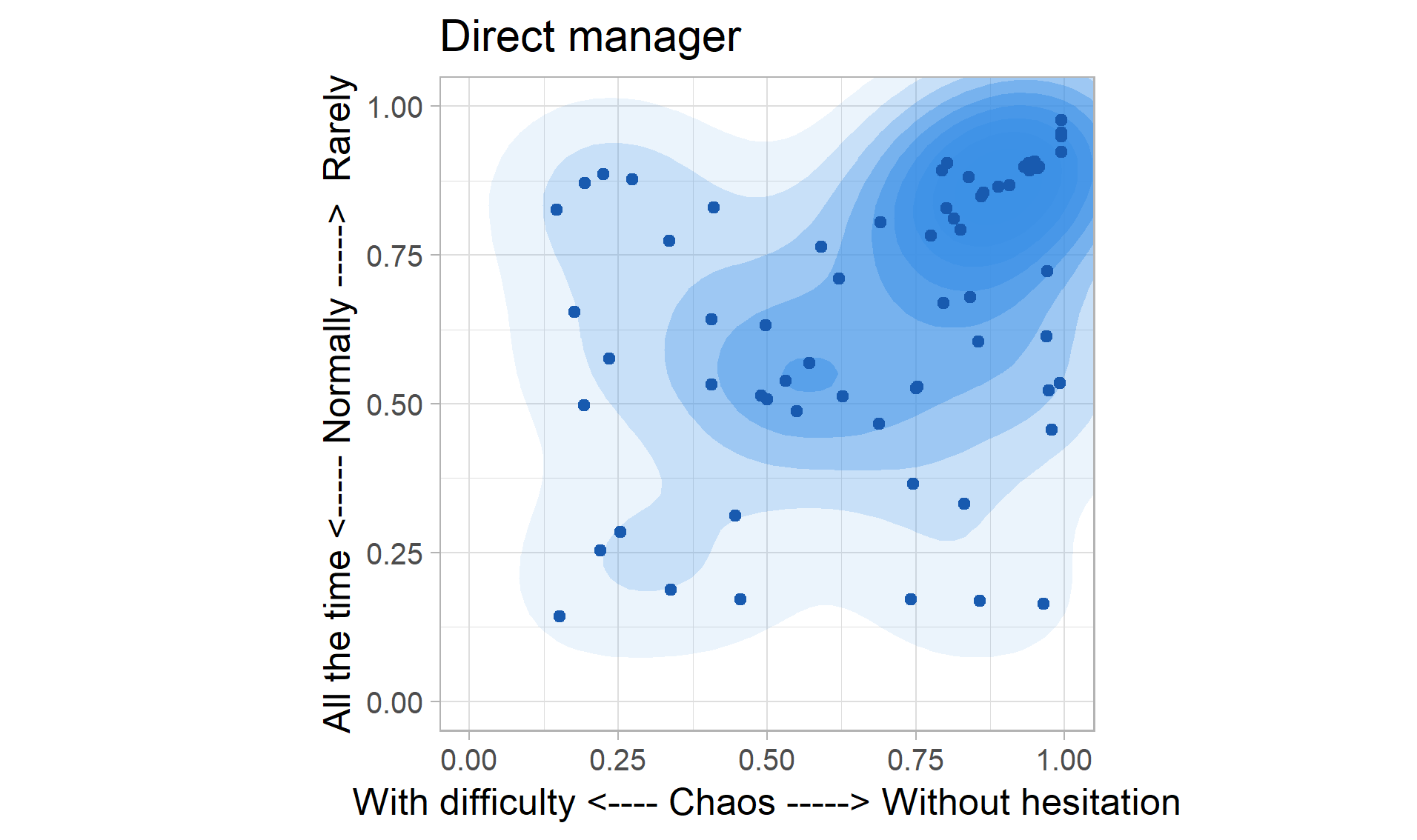

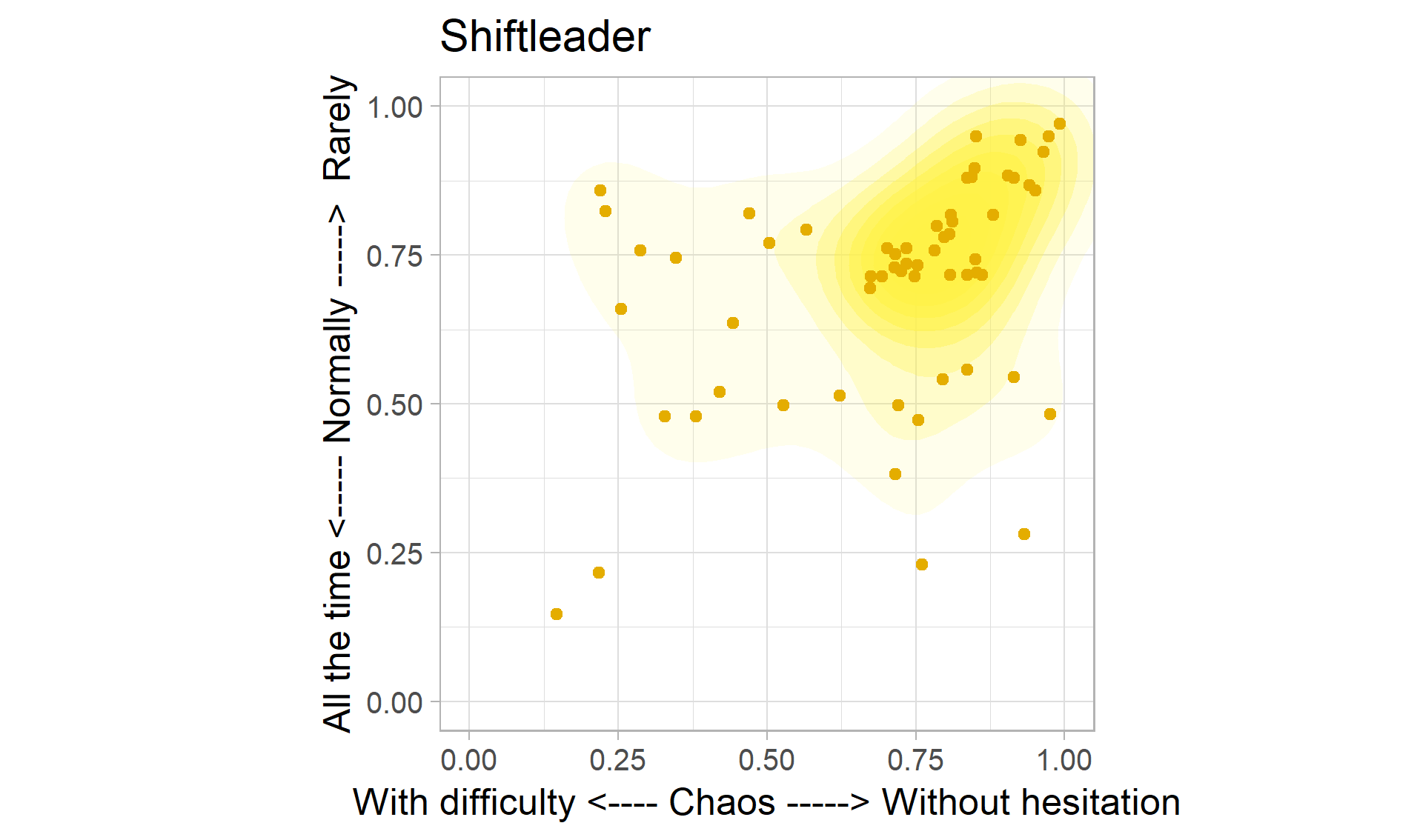


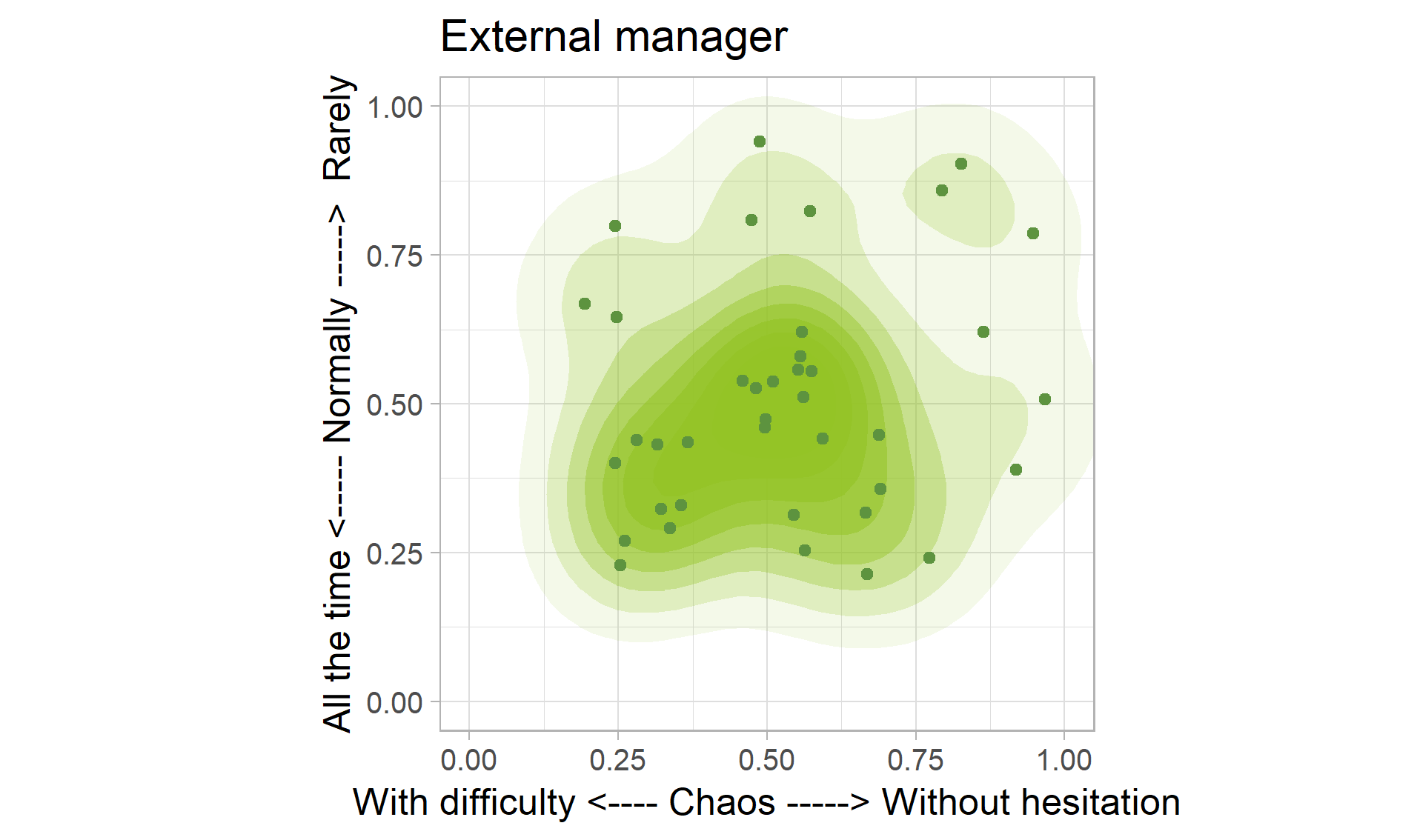

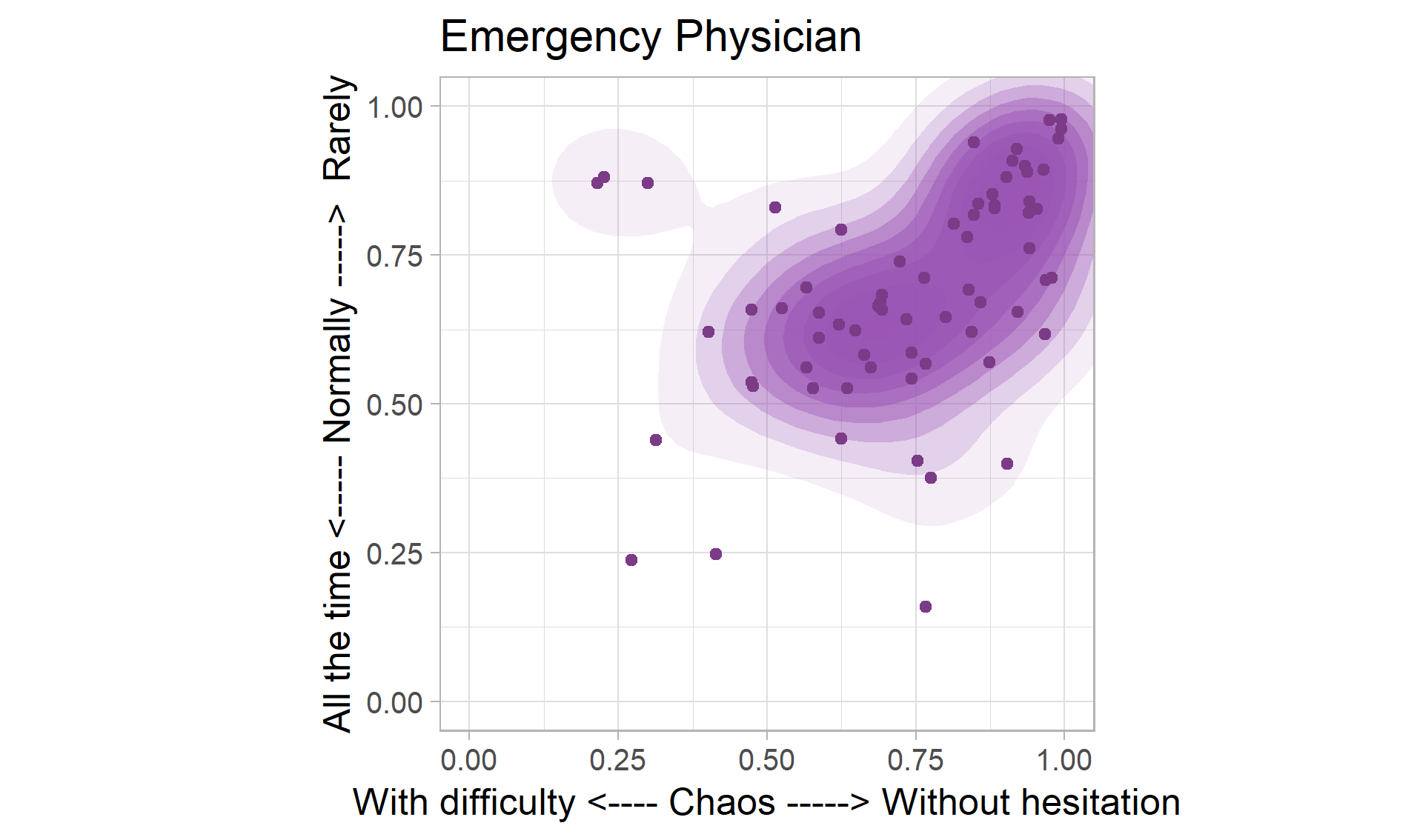


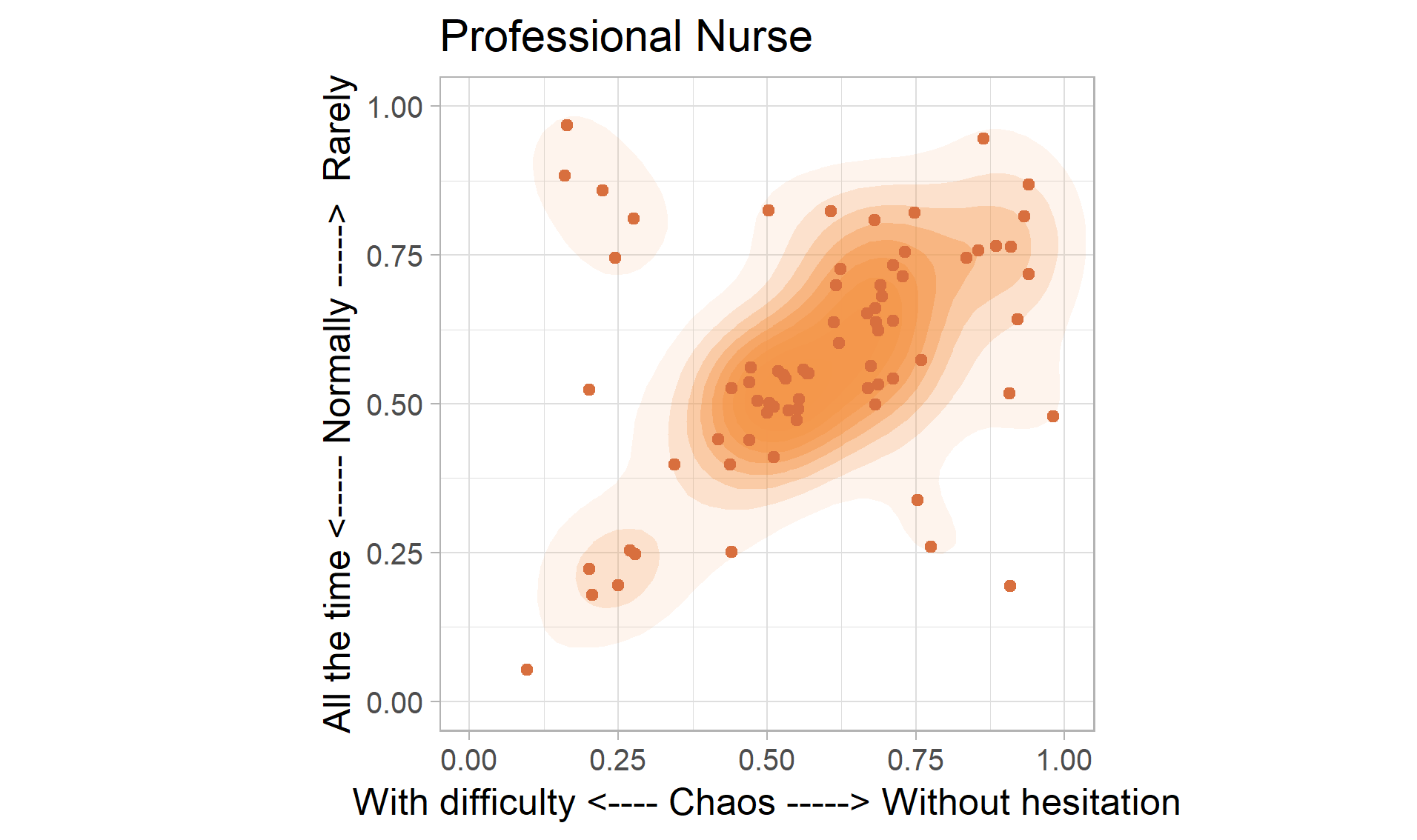

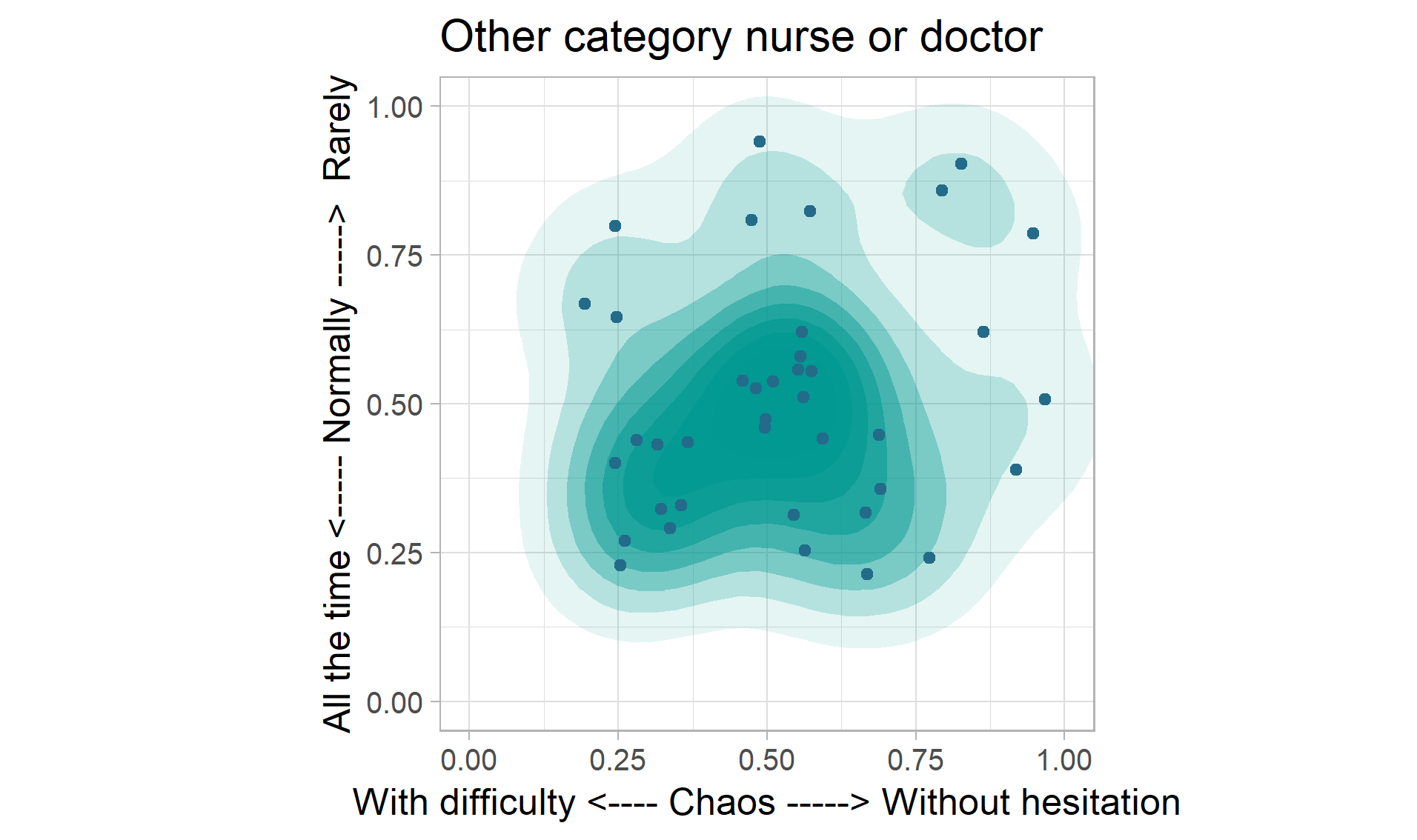


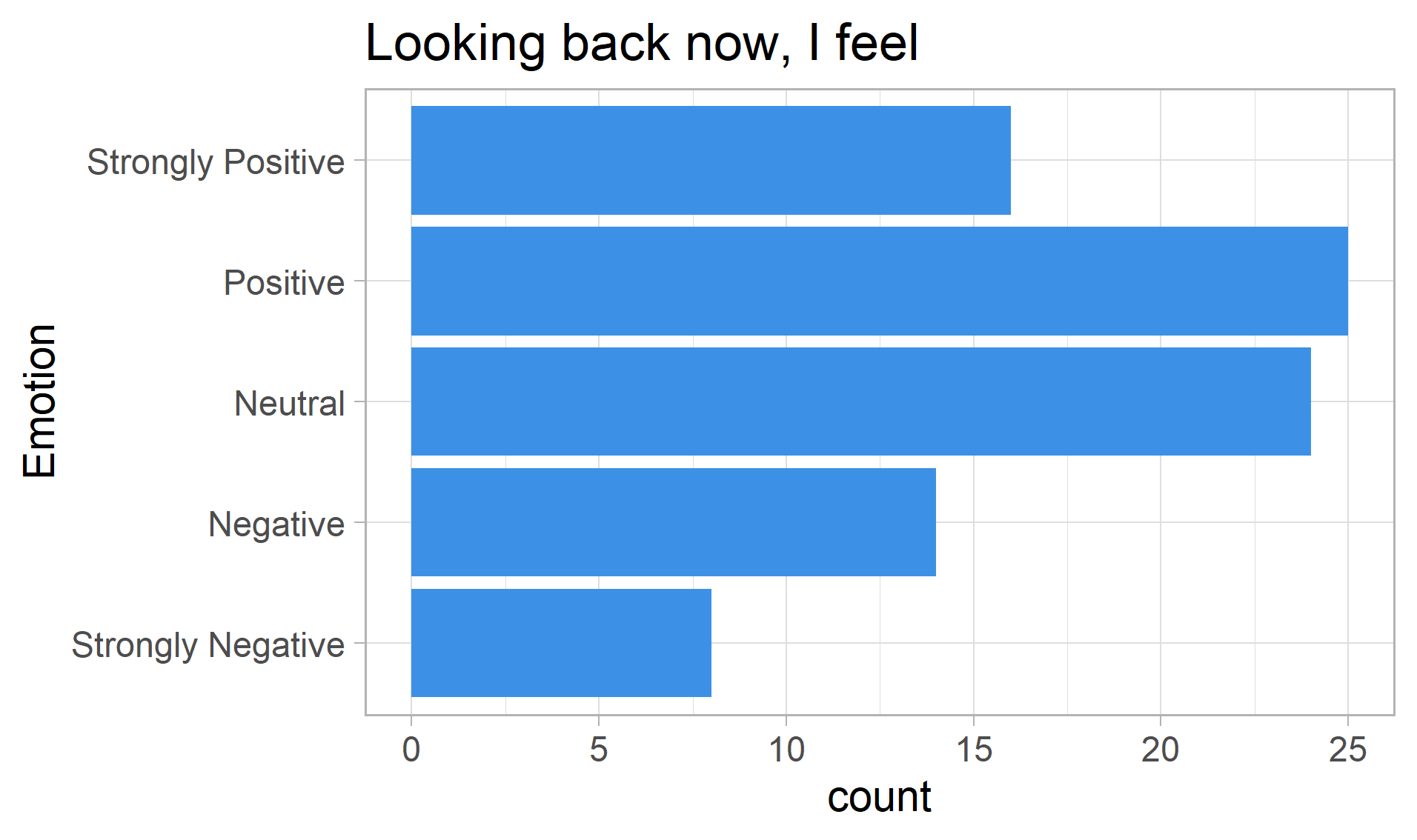


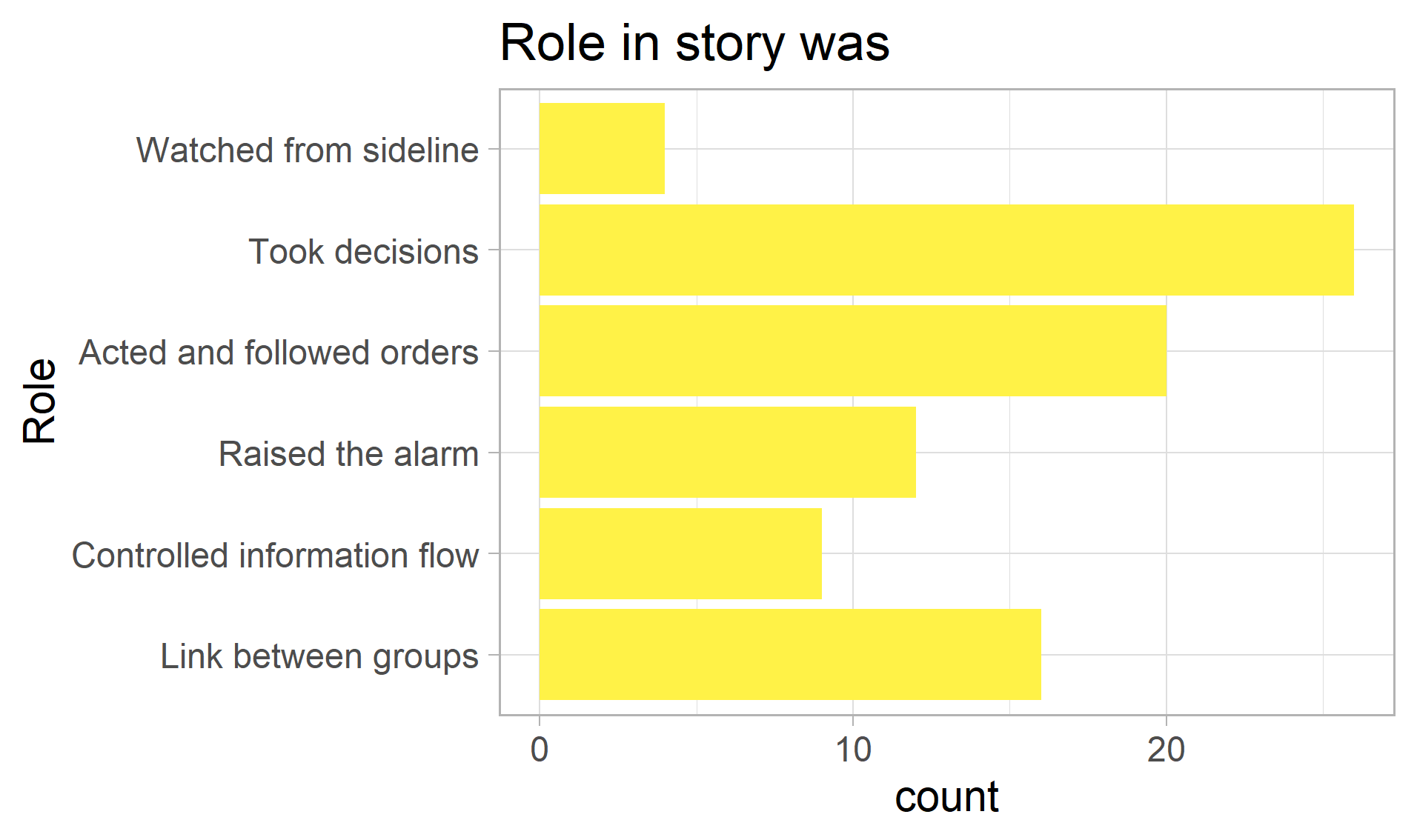


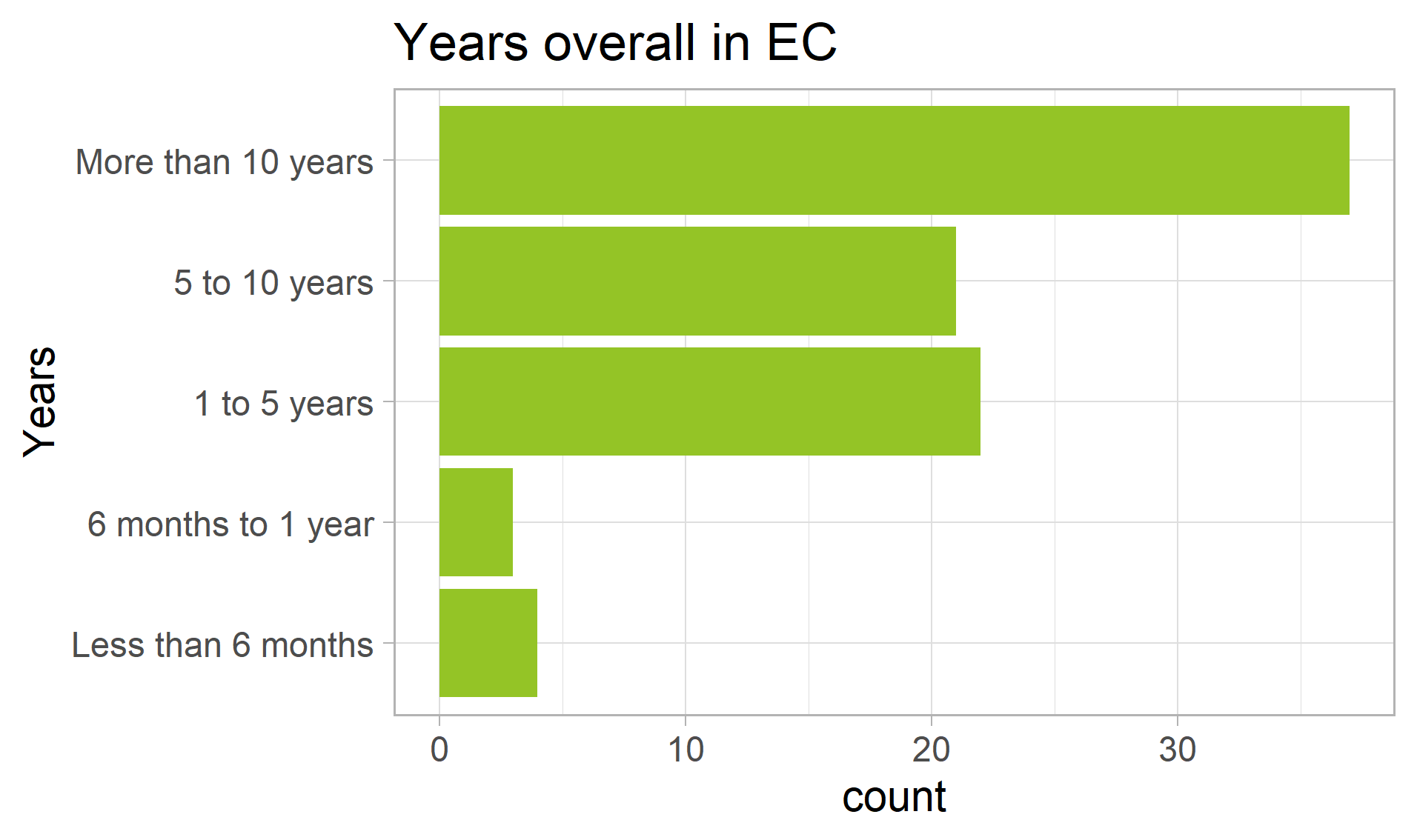


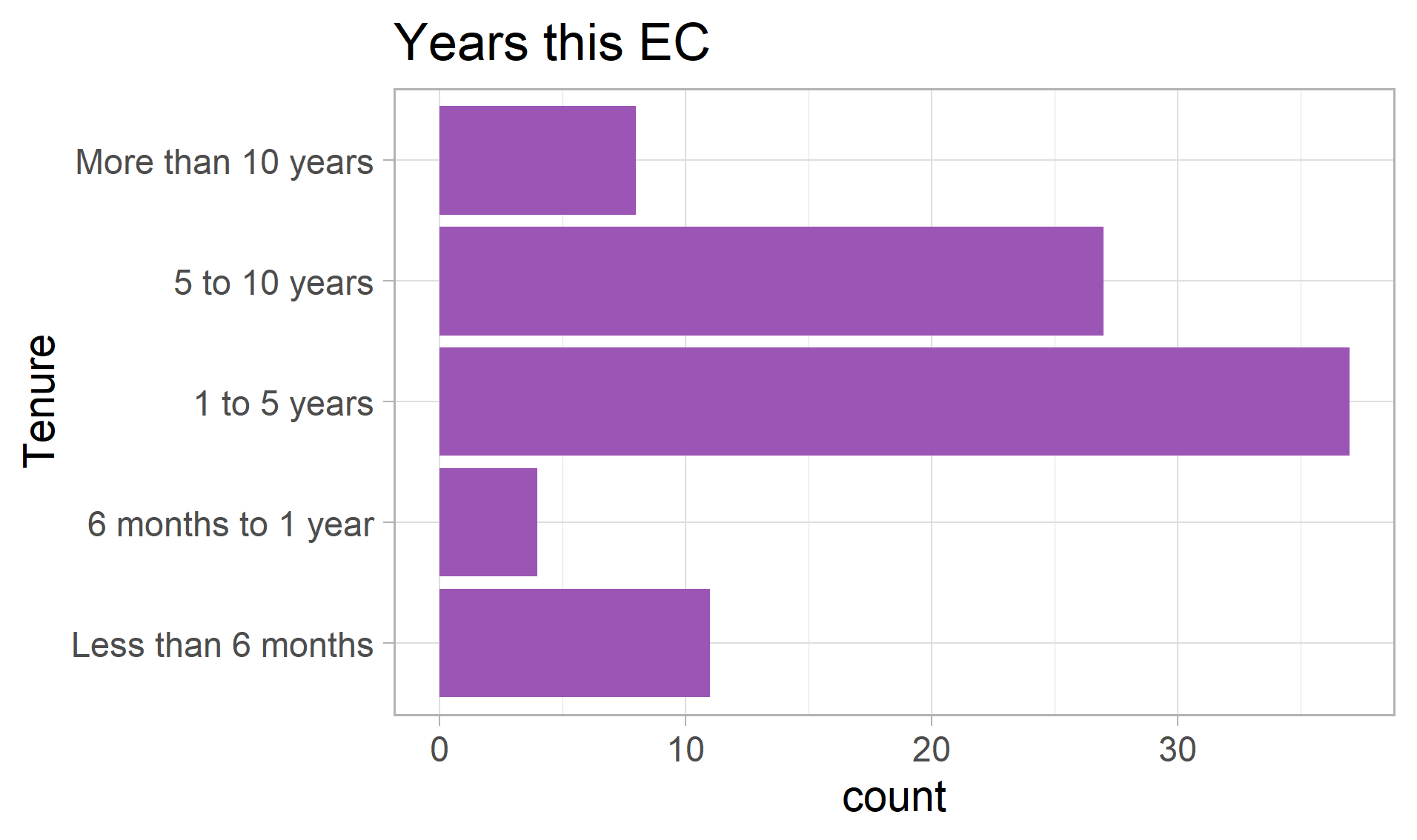


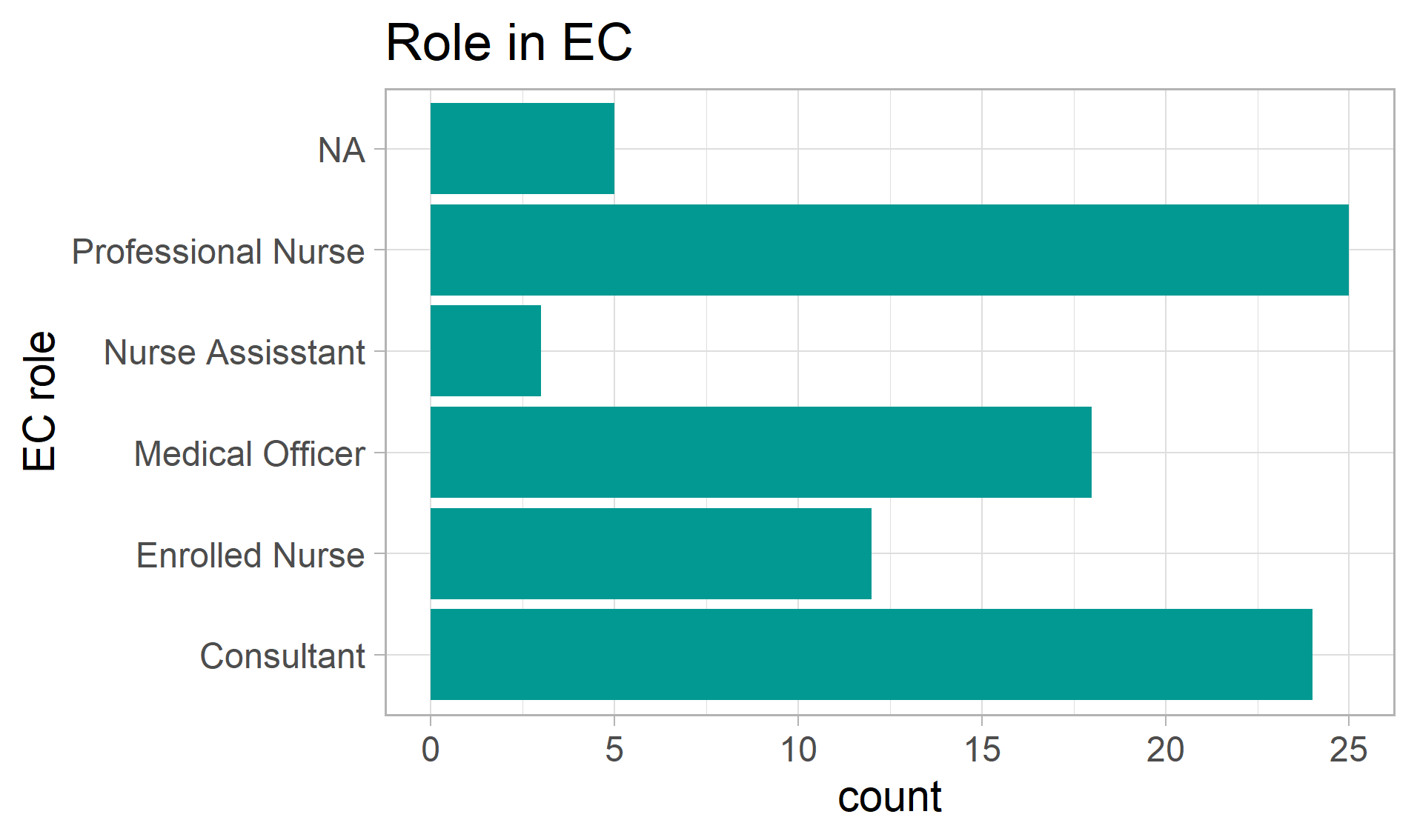


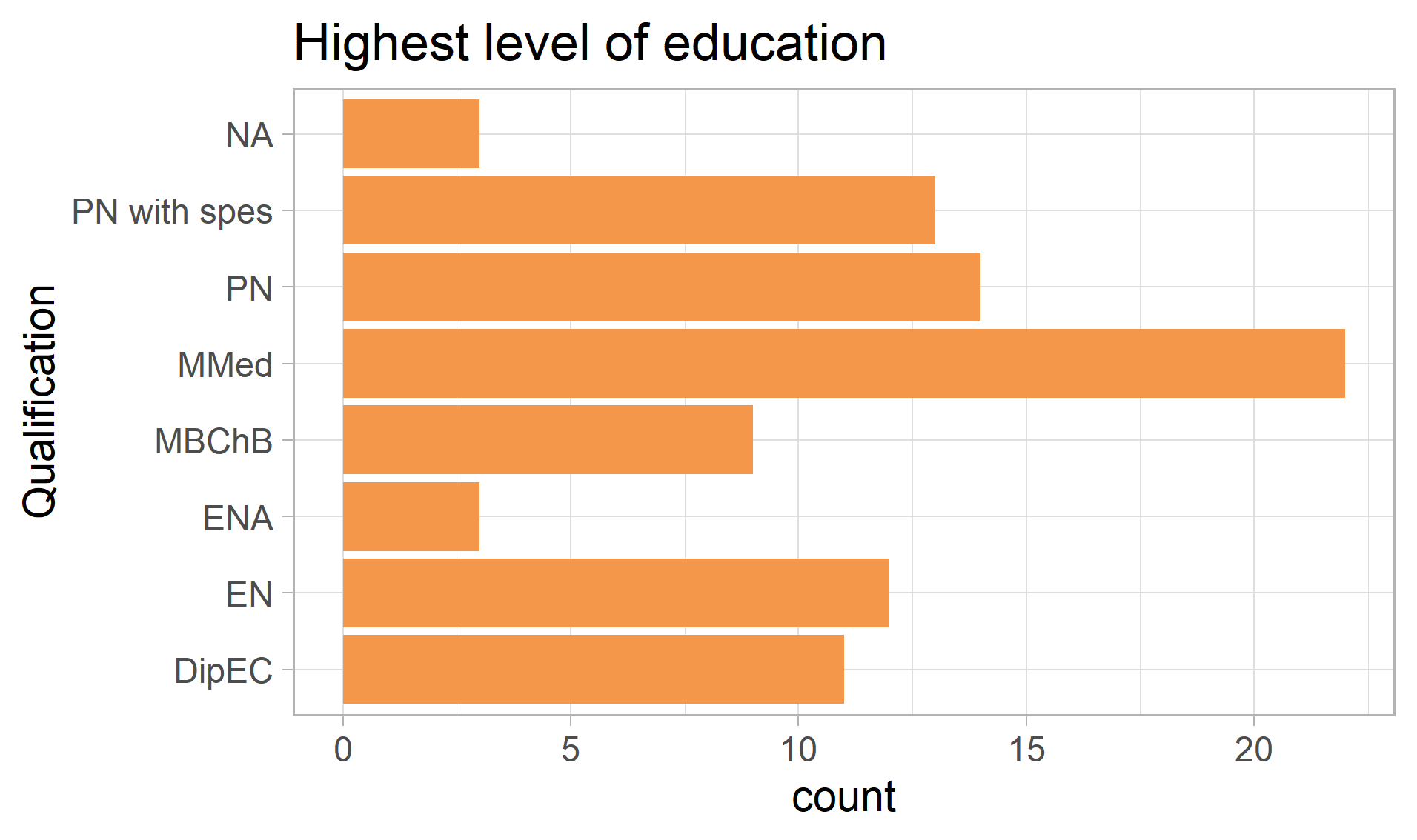

Supplement: S2 Appendix — (DOCX) [file pone.0282307.s002.docx]
